# Beyond what meets the eye: unveiling dynamics of compliance with preventive measures in the COVID-19 era

**DOI:** 10.1186/s12889-026-26347-y

**Published:** 2026-03-04

**Authors:** Sahar Ramazan Ali, Éric Lacourse, Mathieu Pelletier-Dumas, Jean-Marc Lina, Jacques Bélair, Roxane de la Sablonnière

**Affiliations:** 1 Department of Psychology, Université de Montréal, Montréal, Canada; 2 Department of Sociology, Université de Montréal, Montréal, Canada; 3 Department of Electrical Engineering, École de Technologie Supérieure, Montréal, Canada; 4 Department of Mathematics, Université de Montréal, Montréal, Canada

**Keywords:** COVID-19, Compliance, Preventive public health measure, Social change, Latent trajectories

## Abstract

**Background:**

Previous longitudinal studies identified variability in compliance with COVID-19 preventive measures, noting the heightened sensitivity of the least compliant groups to situational factors like easing restrictions. However, they overlooked other forms of variability inherent in compliance behaviour. Hence, we investigated compliance with social distancing and staying-at-home measures, and its dynamic nature, along with its association with social and individual factors.

**Methods:**

Data from a longitudinal study involving 1984 Canadians across six time points from April 2020 to July 2020 were analyzed. Compliance levels were assessed through self-reported items, alongside social and individual factors like trust in Canadian health services and in the Prime Minister, perception of social norms, and subjective health literacy.

**Results:**

Latent Class Growth Analysis revealed three compliance trajectories during mandatory and lifting measures periods: “Low and constant” (shifting to “Low and decreasing” during lifting measures), “High and decreasing,” and “High and constant” for both preventive measures (social distancing and staying-at-home). Most Canadians belonged in the “High and decreasing” trajectory during the mandatory and lifting measures periods: 53.68% and 54.89% for social distancing; 51.71% and 53.48% for staying-at-home. Subjective health literacy consistently predicted membership to trajectories of compliance with preventive measures. Transition movements between trajectories mostly showed stability (between 86.83% and 91.55% for social distancing; between 80.24% and 88.69% for staying-at-home). Two factors, namely perception of provincial norms and trust in Canadian health services, were moderate predictors of transitions toward higher compliance trajectories for both preventive measures during the mandatory and lifting measures periods.

**Conclusions:**

Our study not only reaffirms variability in compliance patterns within the lowest compliance group but also unveils variability among higher compliance groups, notably in trajectory transition movements. Subjective health literacy consistently emerged as a strong indicator of trajectory membership, while perception of provincial norms and trust in Canadian health services moderately influenced trajectory change.

**Supplementary Information:**

The online version contains supplementary material available at 10.1186/s12889-026-26347-y.

## Introduction

Research related to COVID-19 saw an exponential growth following the World Health Organisation’s announcement on the severity of the virus outbreak, upgrading its classification to a pandemic. Researchers from diverse fields were mandated by government agencies to answer society’s pressing questions in response to the COVID-19 pandemic, whether they were related to the virus’ transmission modes or to the communities’ adaptive capacities. One of the more prominent themes in the novel COVID-19 literature is compliance to preventive measures, namely hand washing, mask wearing, social distancing, and staying at home. Early on, during the first wave of the pandemic, studies reported that a small minority (between 5% and 12%) did not comply with governmental guidelines, furthering the need to explore the reasons behind this transgressive behaviour [1–3]. These studies evaluated compliance levels to preventive measures as a stable phenomenon without considering transition movements between levels of compliance as the pandemic situation evolved. Furthermore, several social and sociodemographic factors were identified as probable reasons behind non-compliance to preventive measures, but these factors were only examined regarding their relationship with compliance levels and not change in compliance levels [4–7]. Hence, the current study represents a continuation of a longitudinal investigation initiated by Courdi et al. [2] with a representative Canadian sample (*N* = 3617). The primary aim of the earlier study was to identify trajectories of compliance with social distancing, contacts limitation, and mask wearing throughout the first year of the pandemic and its relationship with level of understanding and source of information. This study goes beyond identifying compliance trajectories with preventive measures by also examining transitions between trajectories, particularly during periods of contextual change, such as the shift from mandatory to lifted measures. Additionally, we explore the association between compliance patterns and factors influencing these transitions during the first wave of the pandemic.

The global situation during the early months of the COVID-19 pandemic was critical, with the virus spreading rapidly across all countries, disregarding geographical boundaries. Within two months of reporting the first case, the World Health Organization documented approximately 20,000 cases in March 2020 [8]. As a result, governments worldwide implemented preventive measures to mitigate the spread of the virus, as vaccines were still in the development phase. Essential preventive measures were classified into two categories: individual and community-based measures [9]. Individual measures encompassed contact limitation (staying-at-home and social distancing), respiratory and hygiene etiquette, indoor ventilation, and surface disinfection [9, 10]. Meanwhile, community-based measures comprised the closure of non-essential businesses and restrictions on the number of people in a setting [9, 10]. Given that individual measures relied on voluntary compliance from the population, it became more pertinent to delve into these compliance behaviours rather than focusing on forced directives, such as business closures [11]. However, the effectiveness of each individual preventive measure was not equivalent but hinged on its ability to limit the spread of the virus [10]. For instance, limiting contacts by staying at home was regarded as the most effective measure as it prevented individuals from coming into physical contact with others, thereby significantly reducing the risk of transmission [10]. If limiting contacts by staying-at-home was impossible, it was recommended to implement physical barriers, such as practicing social distancing and wearing masks. Notably, social distancing took precedence as a physical barrier measure over mask wearing, because it was specifically designed to restrict interactions between individuals, thus also decreasing the likelihood of infection [10]. Hence, studies suggested focusing on staying-at-home and social distancing as measures of interest when studying compliance during the pandemic, as they significantly influenced transmission rates [12, 13].

Longitudinal studies on compliance behaviour with social distancing and staying-at-home measures revealed notable variations in compliance levels during the COVID-19 pandemic. Indeed, a study conducted in the United Kingdom with 50,000 participants during the first year of the pandemic found four distinct longitudinal patterns of compliance: *Class 1* (32.80%) with high and constant compliance, *Class 2* (28.66%) with high and fluctuating compliance, *Class 3* (23.98%) with medium high and fluctuating compliance, *Class 4* (14.56%) with low and decreasing compliance [1]. Other longitudinal studies examining trajectory compliance have consistently identified a similar number of compliance groups and reported a decline in compliance, particularly evident in the lowest compliance group during the initial months of the pandemic, prompting further investigation [2, 3, 14].

The decrease in compliance with preventive measures, specifically to social distancing and staying-at-home measures, was first observed at the end of the first pandemic wave (Spring 2020) as highlighted in previous literature [1, 3, 14]. In the Canadian pandemic context, this first pandemic wave spanned from March 2020 to July 2020 [16, 17]. During that first wave, Canadians experienced tremendous changes, such as being required to comply with governmental recommendations under the threat of sanctions [18]. As the COVID-19 situation improved with fewer reported cases, provincial governments gradually lifted mandatory preventive measures, permitting individuals from different households to gather at home, for instance [15, 16]. Hence, two noticeable periods emerged during the first wave of the pandemic in Canada: mandatory measures period and lifting measures period. With slight variations among the provinces, the mandatory measures period commenced in March 2020, and the lifting measures period began toward the end of May 2020 [15–17]. Whereas the previously mentioned longitudinal studies successfully identified variations in compliance levels during that first pandemic wave [1–3, 14], they were unable to explore the effect of the easing of measures on membership in compliance trajectories. For example, in Courdi et al. [2], the authors focused on identifying compliance trajectories during the first year of the pandemic, without considering the changing context, which limits our understanding of behavioural dynamics. Our study innovatively addresses this gap by not only identifying compliance trajectories but also examining whether individuals transitioned trajectories between periods of mandatory and lifting measures. Therefore, two questions arise in relation to this gap: (1) Are there different trajectories of compliance with regards to social distancing and staying-at-home measures during both periods, and (2) did individuals persist in similar compliance trajectories during the first wave of the pandemic?

To examine the presence of different trajectories and shifts in trajectory membership between the mandatory and lifting measures periods, it is recommended to conduct a Joint-Trajectory Analysis [18–20]. An extension of the Latent Class Growth Analysis (LCGA), the Joint-Trajectory not only identifies distinguishable trajectories of response over time but also enables the estimation of probabilities of co-occurrence or continuity in two distinct yet related behaviours [18, 19, 21]. It has been widely employed in developmental research to investigate how trajectories of a behaviour at an early age can serve as precursors for maladaptive behaviour later in life [21–23]. For example, in Côté et al. [21], the researchers aimed to examine the relation between trajectories of physical aggression at an early age and trajectories of indirect aggression later. They reported that most children exhibiting a low trajectory of physical aggression were consistently paired with a low trajectory of indirect aggression later on, indicating stability in aggression levels. On the other hand, among children initially showing higher levels of physical aggression, a shift toward a trajectory of high indirect aggression was observed. A comparable study design in the context of compliance to preventive measures has not been identified in the literature, leaving a gap in our understanding of behaviour dynamics during a crisis such as the COVID-19 pandemic (i.e., when a key event occurs such as lifting of measures). Such a study could aid governments in targeting behaviours susceptible to change as situations evolve and identifying associated risk factors.

### Factors

During the COVID-19 pandemic, researchers sought to identify factors of compliance with preventive measures, aiming to offer valuable insights for government agencies in refining their intervention strategies. Numerous social (e.g., social norms, trust, risk communication) and individual characteristics (e.g., subjective health literacy and perceived self-efficacy) were identified as consistent factors of compliance with preventive measures like social distancing and staying-at-home [5–7, 9]. However, these factors were examined in the context of adopting a compliant behaviour rather than sustaining or reinforcing it as the situation evolves. This distinction is significant, as different processes and interventions come into play for both behaviours, as highlighted in the Kwasnicka et al. review [24]. Hence, outlined below are the factors anticipated not only to forecast trajectory membership but also to predict changes in trajectories between mandatory and lifting measures periods.

Between January 1 st and June 30th, 2020, a total of 23,634 scientific articles on the topic of COVID-19 were documented globally [25]. Indeed, media outlets and the scientific community generated new information at an unprecedented rate, prompting the World Health Organization to label this phenomenon as an “infodemic” [26]. The term “infodemic” is specifically employed in the context of a disease outbreak, referring to an excessive amount of information that can be harmful if misleading [26]. This excess of information can contribute to the adoption of risk-taking behaviours that directly impact health and may exacerbate the course of an outbreak [26, 27]. Therefore, the ability to comprehend, utilise, and critically assess new health information, which translates to the three dimensions of health literacy, becomes crucial in the face of an information overload [28]. In Canada, the lack of health literacy is a well-documented concern for public health officials [29]. Reports indicate that nine million people have limited literacy skills [30], with over half of Canadians reading below a high school level [30]. These statistics are alarming considering the prejudicial impacts of low health literacy on health outcomes, like higher mortality rates and more hospitalisations [28]. On the other hand, high health literacy promotes better awareness of health issues and their consequences, facilitating the adoption of adaptive health behaviours, such as preventive measures during the pandemic [27]. A team of researchers from France investigated the impact of health literacy on compliance with social distancing measures and discovered a positive association, indicating that higher health literacy was linked to increased compliance with social distancing [4]. Other studies also confirmed the pivotal role of health literacy in fostering the adoption of preventive behaviours in the context of the COVID-19 pandemic [31, 32]. In addition to facilitating the adoption of new health behaviours, health literacy also acts as a facilitator when individuals need to better adapt to evolving situations, like the gradual easing of governmental restrictions [27]. As Canadians transitioned to the lifting measures period, individuals with high health literacy were better adjusted to the changing environment, hence expected to more likely maintain high compliance levels or transition to higher compliance responses due to their critical assessment of the still-present virus’s dangerousness.

As the pandemic unfolded, practices once considered normal in social settings, such as shaking hands, were strongly discouraged and replaced by behaviours like social distancing and staying-at-home as much as possible to limit the spread of the virus. Therefore, adopting these preventive behaviours to protect oneself and others became the prevailing social norms during the pandemic, establishing a set of expectations for the new normalcy [33]. These expectations were based on *others*’ behaviours (descriptive norms) or *others*’ attitudes and beliefs (injunctive norms), *others* being the ingroup. In the existing literature, there is a consensus among researchers that descriptive norms are more influential in prompting changes in health behaviours compared to injunctive norms due to the high conformity pressure associated with them [34–36]. A comparable conclusion was reached in a COVID-19 compliance study conducted in Spain [5]. The authors noted that the perception of descriptive norms was the most important factor of compliance with social distancing measures, followed by trust in science, perceived effectiveness of measures, and finally, perceived risk of infection. Furthermore, a high perception of descriptive norms is not only more likely to induce the desired behavioural change but also to foster sustainable changes in behaviour, possibly due to the norm internalisation process that is immune to situational factors like the lifting of preventive measures [37]. Thus, individuals who initially reported a strong perception of descriptive norms should be expected not only to comply strongly with preventive measures, as indicated in Cabrera-Álvarez et al. [5], but also to continue exhibiting high levels of compliance as the pandemic situation evolves. Finally, when referring to descriptive norms, the *others* become crucial in navigating new expectations [7], since they represent individuals from the same group. In the Canadian political landscape, the *others* could be Canadians in general or the residents of the same province [33]. To our knowledge, no study has made this distinction in regard to compliance with preventive measures, which would help governments target relevant national and provincial interventions.

During the pandemic, some individuals were confined in small living arrangements, while others were unable to work remotely. In both instances, situational factors posed obstacles to compliance with preventive measures, specifically social distancing and staying-at-home, which can hinder perception of control and ultimately perceived self-efficacy. Perceived self-efficacy reflects the belief in one’s capability to modify health behaviours by taking personal actions [38]. It consistently emerges as a significant individual factor in various health behaviour theories, such as the Health Belief Model [39], Reasoned Action approach [40], and Protection Motivation Theory [41]. In the Health Belief Model, for example, the likelihood of adopting a preventive health behaviour is dependent on having greater confidence in one’s ability to apply that health behaviour, which amounts to higher perceived self-efficacy [39]. Additionally, individuals with a high sense of self-efficacy tend to invest more effort and demonstrate greater persistence compared to those with low self-efficacy [38]. In the face of obstacles, like living in small arrangements and the impossibility to work remotely, the former rebound more quickly and maintain health behaviours. On the other hand, studies report that a low sense of self-efficacy can have damaging effects beyond decreasing compliance to health behaviours, like higher risk of depression, anxiety and helplessness [42]. In the context of the COVID-19 pandemic, this individual factor was studied in relation to compliance with preventive measures such as social distancing and staying-at-home. In a longitudinal study conducted in China [6], the importance of perceived self-efficacy and perceived barriers was highlighted as crucial factors in adopting and sustaining compliance with physical distancing measures over a span of three months, even in the presence of stressors such as the lifting of preventive measures. Given these results [6], individuals with high perceived self-efficacy are expected not only to initially exhibit high compliance behaviour but also to sustain or transit to these high levels of compliance, even during the transition between mandatory and lifting measures periods.

During the initial months of the virus outbreak, many individuals questioned the government’s ability to effectively manage the pandemic situation, while others challenged the public health institutions to provide reliable information. The lack of trust at both levels, government and public health services, has been widely documented as a strong factor of defiant health behaviour [43–45]. Trust in the government (such as a Prime Minister, a President, a local council) is akin to supporting and being confident about public directives [46]. To preserve a functional democracy during an economic or social crisis, a minimum level of trust in the government is necessary, as it would be easier to implement directives in the population if they believed in the competence of governmental institutions to deal with the crisis [47]. Trust in public health institutions involves being confident in the results produced by the healthcare and scientific community and their ability to elaborate effective social and economic policies [48]. Indeed, when new issues or problems emerge, individuals, lacking the resources to understand these complex problems, must come to trust public health institutions to make the most appropriate decisions [48]. The literature indicates that individuals with higher trust in public health institutions, such as health services in Canada, were found to be more open to acquiring new knowledge related to COVID, facilitating their adaptation to novel information, and ultimately aiding in navigating an evolving situation such as the easing of measures [49]. In light of previous findings, it is expected to find similar results in the Canadian landscape, where individuals who expressed high levels of trust in both the provincial Prime Minister and in Canadian health services exhibited greater compliance with preventive measures.

At the onset of the health crisis, Canadian provincial governments implemented region-specific public policies, particularly concerning preventive measures. This decentralised approach offers advantages, such as increased flexibility and innovation. However, solely relying on this governance model does not ensure satisfactory outcomes, as evidenced by varying death and case numbers across provinces during the pandemic [17]. According to a report from the Organisation for Economic Co-operation and Development [50], the effectiveness of crisis management lies in fostering coordination between different government levels and clearly defining their respective roles. Such coordination can enhance coherent communication between provincial and federal authorities and result in clearer dissemination of government messages at all levels [50]. Effective communication, grounded in principles of coherence and clarity during a social crisis, is more likely to motivate individuals to support public policies, including preventive measures [51]. As validated by Pelletier-Dumas et al.[52], a heightened perception of clarity and coherence in the government’s communication strategy was linked to increased compliance with preventive measures. Given the dynamic nature of the pandemic, clear and coherent communication became increasingly crucial as Canadians navigated the lifting measures period, marked by the uncertainty and novelty of the situation. Hence, individuals perceiving coherent and clear messages from both governance levels (federal and provincial) will be more likely to comply with preventive measures during the mandatory and lifting measures periods and transition or maintain high compliance behaviour during the transition period.

In the literature, some studies considered the effect of socio-economic characteristics such as age, gender and level of education. It has been shown that older people are more likely to comply with preventive measures [53, 54]. Women and university-level graduates seem to follow the same trend, i.e. a higher level of compliance with preventive measures [55–58].

The current research aims to identify patterns of compliance with preventive measures, like social distancing and staying-at-home, during the mandatory measures and lifting measures periods in Canada. This project also investigates the dynamic nature of trajectory change as Canadians transitioned between periods. Finally, we examine the factors’ association with trajectory and trajectory change to further our understanding of the dynamic nature of compliance during a sanitary crisis.

### Hypothesis

The first objective attempts to identify trajectories of compliance to preventive measures during the first pandemic wave, which can be divided into 2 distinct periods. The first period is associated with the mandatory imposition of measures (April 2020 - May 2020), while the second period represents the lifting of measures (May 2020 - July 2020).

1. In response to this objective, we anticipate that different trajectories of compliance will be identified during both periods (March 2020 - May 2020/May 2020 - July 2020).
2. Also, based on previous studies [1–3, 14], we suggest that the majority of Canadians will be grouped into trajectories of high levels of compliance during both periods of the first wave.

The second objective relates to describing the transitions between both periods of the first wave in terms of group membership in compliance. It entails quantifying the percentage of Canadians transitioning from one group of compliance to another between periods (April 2020 - May 2020/May 2020 - July 2020).

1. We expect to find some Canadians transitioning from one group of compliance to another between periods.
2. We also expect that the majority of Canadians will preserve similar levels of compliance between both periods.

The last objective aims to identify factors of compliance at baseline in April 2020 and during the transition period, which refers to the transition between the first and second periods of the first wave.

1. At baseline, we expect that there will be differences in compliance with preventive measures due to variables like perception of social norms, level of health literacy, perceived self-efficacy, levels of trust in Canadian health services and in the provincial Prime Minister. High compliance trajectories at baseline (April 2020) will be associated with higher levels of perception of social norms, higher levels of subjective health literacy, more social support, higher perceived self-efficacy and more trust in the provincial Prime Minister and in Canadian health services compared to the modal trajectory of compliance.
2. During the transition period, Canadians who initially recorded higher level of perceptions of social norms, higher levels of subjective health literacy, higher perceived self-efficacy and more trust in Canadian health services will preserve high and constant levels of compliance to preventive measures. Trust in the provincial Prime Minister will not be associated with transition movements between trajectories of compliance, as its impact on compliance to preventive measures is not consistent in time [7].

## Methods

### Sample

The current study is part of a larger survey titled “COVID-19 Canada: The end of the world as we know it?“. A similar description of the survey used in this study can be found in Courdi et al. [2]. The underlying longitudinal survey, central to this project, was executed in partnership with the polling firm Delvinia, utilising the AskingCanadians survey panel, which comprises an extensive database of over one million Canadians. This longitudinal survey encompassed twelve time points spanning a duration of 2 years, from April 2020 to April 2022. However, for the purposes of this study, we focus exclusively on the initial six time points, extending from April 2020 to July 2020, which represents the first pandemic wave. Time was encoded by weeks to accommodate the varying intervals between each time point (see Supplementary Materials). In terms of sampling, the project “COVID-19: the end of the world as we know it?” accounted for 3617 participants in the first measurement time. In the current study, the final sample encompassed 1984 participants. This sample is non-probabilistic, employing the weighted quota method for participant selection. Upon further analysis of the representativeness of the sample, the researchers found that the sample was representative in terms of household composition, employment rate, immigration status. However, when the sample is compared with other socio-demographic characteristics, it is less representative for certain groups, such as Francophones, Canadians with lower levels of education and First Nations [59]. Attrition was observed from the second wave of data collection onward, with an average attrition rate of 43.35% (standard deviation = 6.12%) for measurement time points two to twelve, ranging from a minimum of 34.50% to a maximum of 53.77%. For young people, the attrition rate seems to have been greater over time. To limit the effect of differential attrition according to certain individual characteristics, we incorporated weights based on demographic characteristics (e.g., age, gender, province of residence, household income, etc.) in the modelling of trajectories. Finally, in this project, we used full information maximum likelihood estimation in modelling trajectories of compliance with preventive measures [60].

LCGA typically necessitates a minimum of two measurement time points for each period (mandatory and lifting measures periods). Therefore, participants who did not provide a minimum of two responses during each period were excluded. For example, if a participant missed two or more responses within a specific period, they were excluded from the final sample. Only participants who answered at least twice for each evaluated period were included. This resulted in a final sample size of 1984 participants.

A preregistration of this study can be found here: 10.17605/OSF.IO/R276S. An initial analysis was conducted using the provided computation of variables, but multicollinearity issues were identified, necessitating additional attention. As a result, the format in which factors are presented in the subsequent sections reflects the final, resolved format. For more details, see supplementary materials.

### Compliance to preventive measures

Participants self-reported their compliance levels to social distancing and staying-at-home measures from April 2020 to July 2020 at six measurement times. They answered the following statements on a scale of 1 to 10 (never to always): “Currently, how often do you do the following? (1) Maintain a distance of at least two metres (about two arm’s lengths) from others when I am not at home; (2) stay home as much as I can. Since this study is the continuation of the Courdi et al. study (2023), we used the same items to assess compliance behaviour with social distancing and staying-at-home. For the multinomial logistic regression analysis, the trajectories were transformed as binary outcomes (0 = stayed in a low trajectory or shifted to a lower trajectory; 1 = remained in a high trajectory or shifted to a higher trajectory).

### Factors of trajectories and joint trajectories

#### Subjective health literacy

To assess the ability to critically extract and use information in the context of the COVID-19 pandemic, we employed a composite measure that combined seven elements. First, we included subjective literacy items (e.g., “I know what maintaining a social distance means”), which capture individuals’ self-perceived understanding of key public health concepts. Second, we incorporated conspiracy belief items (e.g., “COVID-19 is a hoax”), which serve as an inverse indicator of the capacity to evaluate information critically. Third, we added objective health literacy items (e.g., “Avoiding gatherings with large numbers of people helps prevent the spread of COVID-19”), reflecting respondents’ ability to comprehend and apply factual knowledge. Taken together, this composite was intended to approximate subjective health literacy, that is, the ability not only to understand health information but also to evaluate its credibility and relevance in decision-making. Participants received one point if they provided the correct answer to the statement; otherwise, they were assigned zero point. We summed the points to evaluate the participants’ level of health literacy. Due to the application of planned missingness for this set of questions, not all participants were able to respond to every true and false statement. As a result, participants’ subjective health literacy levels were evaluated using a proportion (number of correct answers/number of statements answered), where a higher proportion score indicated a better subjective health literacy level. The calculation of our subjective health literacy variable was derived from the methodology employed in the study by Montagni et al. [61]. In their study, the authors aggregated participants’ scores to determine the final level of health literacy.

#### Social norms

We interrogated Canadians on their perception of descriptive federal social norms by asking the following question at the first time point: “Most Canadians are following governments’ measures recommendations concerning COVID-19”. We also evaluated their perception of descriptive provincial social norms with this statement at the first measurement time: “In general, [Your province citizens] are complying with the governmental measures”. Both items are on a scale of 1–10, 1 being completely disagree and 10 completely agreeing. Several COVID-19 studies have employed single items to assess perception of descriptive norms [5, 62, 63].

#### Trust

To evaluate Canadians’ levels of trust towards the Canadian health services and the provincial Prime Minister during the COVID-19 pandemic, we used the following question from the survey at the first time point: “How much do you trust each of the following actors to address the COVID-19 crisis?” (1) Canadian hospitals and health services (2) Prime Minister [Name] [Pipe in the name of the Prime Minister]”. The first item refers to trust in Canadian health services, while the second relates to trust in the provincial Prime Minister. Participants answered on a scale of 1–10, 1 distrusting completely and 10 trusting completely.

#### Self-efficacy

This variable refers to the perception of self-efficacy in protecting oneself and loved ones against the COVID-19 virus. It was evaluated with a single item at the first time point: “I do not know what is the best strategy to fight COVID-19”. The item is on a scale of 1–10, 1 being completely disagree and 10 being completely agree. We recoded the variable to establish a positive gradient, wherein a higher score reflects a greater sense of self-efficacy. A similar single item was used in another study [14], where the authors studied the influence of Health Belief factors on compliance levels. In their study, they evaluated the participants’ perception of self-efficacy in regard to the specific behaviour of social distancing. However, in our study, we asked the participants a more general statement since we focused on more than one behaviour of compliance to preventive measures.

#### Clarity

Canadians’ clear understanding of measures was assessed with a single item at the first time point with a 1 to 10 scale (1 = Strongly disagree, 10 = Strongly agree): “In general, I have a clear understanding of the various measures established by Canada’s public health agency”. A similar item was already used in a previous article to evaluate perception of clarity in the message about measures [63].

#### Coherence

The participants’ perception of self-reported coherence towards government messages was assessed with two items at the first time point with a 1 to 10 scale (1 = Strongly disagree, 10 = Strongly agree): “(1) I am confused about the different recommendations coming from the federal and provincial governments and public health agencies, (2) The measures established by Canadian and provincial public health agencies are similar”. The first item was recoded to establish a positive gradient, wherein a higher score reflects a greater perception of coherence. We averaged both items to obtain a final coherence variable. In line with our study, other research endeavours have also employed a self-constructed coherence variable [52].

For determining factors’ association to trajectory and joint-trajectory membership, factors, assessed initially as continuous variables, were coded as binary variables. Indeed, responses falling within the first lowest quartile represented a low score, while responses outside the first quartile were considered as indicating a high score. For instance, Canadians who obtained a result within the first lower quartile that fell or was equivalent to 0.08 were considered to have a low level of subjective health literacy, while others a high level of subjective health literacy. The cutoffs varied depending on the variable, as some factors displayed less or more variation on the distribution. For the analyses, participants were assigned a value of 1 if they self-reported a high level of factors and 0 if they reported a low level. A similar approach was adopted in the Kil and al. study [64] to determine the high and low cutoffs. For more details on the threshold for every variable, see supplementary materials.

### Analyses

We conducted Latent Class Growth Analysis (LCGA), or commonly referred as Group-based trajectory analysis, to discern longitudinal patterns of compliance behaviour to preventive measures, hence regrouping participants with similar trajectories together [18, 20]. Since compliance to preventive measures was assessed as a continuous variable and displayed a normal distribution, a censored normal distribution (CNORM) was employed [66]. In contrast to many other longitudinal methods, LCGA does not necessitate the assumption of homogeneity of variance between time points to be upheld. As suggested by Nagin [18], the Bayesian Information Criterion (BIC) served as our initial guide to determine the most suitable number of trajectory groups. Once we established the number of trajectory groups for the final model, we proceeded to choose the polynomial function for each trajectory—whether constant, linear, quadratic, or cubic. The model with the best fit, as indicated by the BIC, was retained. During model selection, we also considered the participant distribution across trajectory groups, for it is crucial that the trajectories exhibit visually distinguishable patterns. The determination of the polynomial function for each trajectory was further refined based on a significance level (*p-value* < .05). After identifying longitudinal patterns of compliance to preventive measures (social distancing and staying at home) during each period (mandatory and lifting periods), we sought to identify transition movements between trajectories by conducting a Joint-Trajectory analysis, an extension of LCGA [19, 20]. Similarly to the LCGA, the Joint-Trajectory procedure allows for modeliation of trajectories over time. However, the latter distinguishes itself by linking trajectories of distinct but related behaviours. There are two approaches to Joint-trajectory analysis: comorbidity and heterotypic continuity. The first method allows for the estimation of probabilities for simultaneous membership in two distinct but related longitudinal patterns of behaviours, while the second method focuses on predicting the probabilities of the occurrence of trajectories for two behaviours expressed at different periods. In this study, we solely focused on the second approach, as it enabled us to identify transitional movements for trajectories of compliance to preventive measures between the mandatory and lifting measures periods. Finally, we used the RISK function from the PROC TRAJ package in the SAS software to study factors’ association to trajectory membership for both preventive measures during the mandatory and lifting measures periods, while controlling for age, gender and level of education [19, 20]. The modal trajectory was chosen as the reference group, enabling us to discern high and low compliance behaviours from the majority. We also evaluated the factors’ association to transition movements while performing a multinomial logistic regression analysis. The factors were entered as binary variables, and their reference level was low. Given the exploratory nature of this study and limited samples for trajectory movements, we incorporated a lower threshold (*p-value* < .10) to identify trends for potential exploration in future research for this section of the study. All visualisations were generated using the ggplot2 package in R.

## Results

### Compliance trajectories to preventive measures

**Fig. 1:**
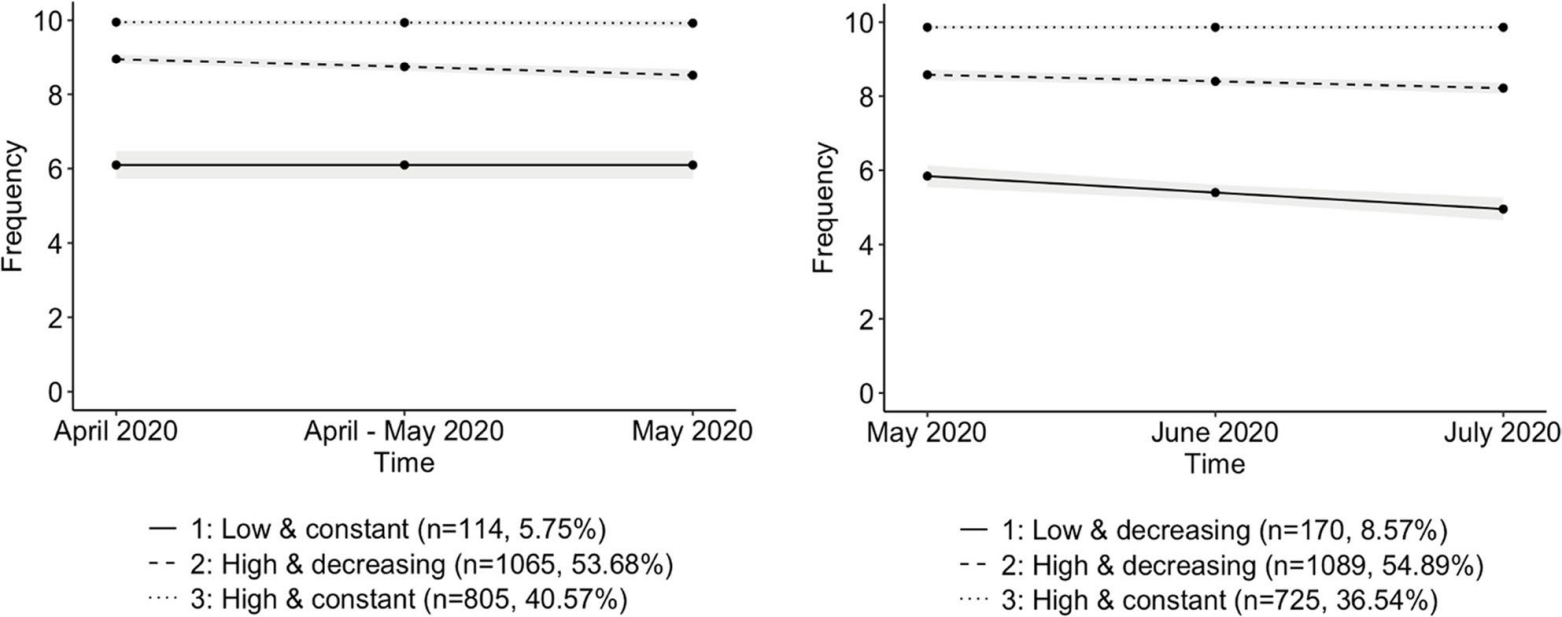
Compliance trajectories to social distancing measure during the first wave of the pandemic (April 2020 - May 2020 and May 2020 - July 2020)

**Fig. 2:**
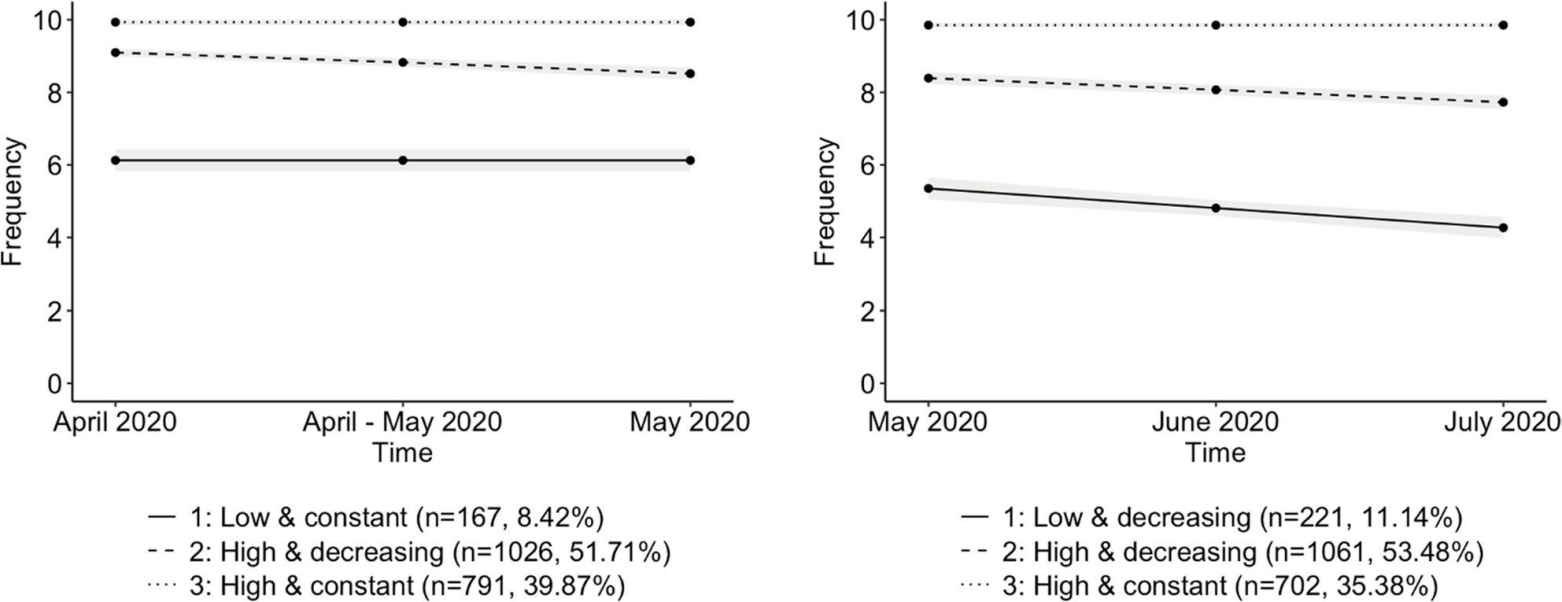
Compliance trajectories to stay-at-home measure during the first wave of the pandemic (April 2020 - May 2020 and May 2020 - July 2020)

In observance of our first objective, we aimed to discern longitudinal patterns of compliance with preventive measures, specifically social distancing and staying at home. Upon conducting group-based trajectory analysis, we determined that three trajectories most accurately characterised our sample’s compliance to both social distancing and staying at home during both periods (mandatory measures and lifting measures). This determination was based on the evaluation of BIC values and the significance of the parameters (see Supplementary Materials). The results presented in Figs. 1 and 2 ; Tables 1 and 2 were derived from participants’ classifications based on the model’s posterior-probability assignment.

Figure 1 depicts the trajectories of the best-fitting models for the social distancing measure during both periods. For the mandatory measures period, we observed three trajectories characterised by constant, linear, and linear parameters (BIC=−7501.67), representing: a *Low and constant* trajectory (*n* = 114, 5.75%); a *High and decreasing* trajectory (*n* = 1065, 53.68%); and a *High and constant* trajectory (*n* = 805, 40.57%). Similarly, for the lifting measures period, three distinct trajectories were identified with linear, linear, and constant parameters (BIC= −8414.28): a *Low and decreasing* trajectory (*n* = 170, 8.57%); a *High and decreasing* trajectory (*n* = 1089, 54.89%); and a *High and constant* trajectory (*n* = 725, 36.54%).

Figure 2 illustrates the trajectories of the best-fitting models for the staying-at-home measure during both periods. For the mandatory measures period, we observed three trajectories characterised by constant, linear, and constant parameters (BIC= −7594.57), representing: a *Low and constant* trajectory (*n* = 167, 8.42%); a *High and decreasing* trajectory (*n* = 1026, 51.71%); and a *High and constant* trajectory (*n* = 791, 39.87%). Similarly, for the lifting measures period, three distinct trajectories were identified with linear, linear, and constant parameters (BIC=−9182.35): a *Low and decreasing* trajectory (*n* = 221, 11.14%); a *High and decreasing* trajectory (*n* = 1061, 53.48%); and a *High and constant* trajectory (*n* = 702, 35.38%).

### Probabilities of heterotypic continuity

Given membership to a trajectory during the mandatory measures period, we aimed to estimate the probabilities of belonging to a trajectory during the lifting measures period, contingent upon the initial membership.

Table 1 displays the conditional probabilities of Canadians’ compliance levels to social distancing during the lifting measures period based on their initial membership during the mandatory measures period. Generally, individuals exhibited stability from one period to another, as indicated by high probabilities along the diagonal. Specifically, 91.22% (*n* = 104) of the sample remained in the lowest compliance trajectories for both periods; 91.55% (*n* = 975) remained in the high and decreasing trajectories, and 86.83% (*n* = 699) remained in the high and constant trajectories. The off-diagonal elements indicate that a small percentage of the population transitioned between periods. For example, 8.77% (*n* = 10) of individuals initially in the “Low and constant” trajectory during the mandatory measures period shifted to the “High and decreasing” trajectory in the lifting measures period. In contrast, 2.44% (*n* = 26) of Canadians transitioned from a “High and decreasing” to a “High and constant” trajectory. A substantial proportion of Canadians (12.92%, *n* = 104) experienced a decline in compliance between periods, moving from a “High and constant” to a “High and decreasing” trajectory. Notably, only two individuals exhibited a substantial decrease in compliance levels, transitioning from a “High and constant” to a “Low and decreasing” trajectory. No transition movements were identified from the “Low and constant” to the “High and constant” trajectories.

**Table 1:** Percentage (and Number) of individuals in each trajectory for social distancing measure, by period

	Lifting measures period		
Mandatory measures period	*Low and decreasing*	*High and decreasing*	*High and constant*
*Low and constant*	**91.22 (104)**	**8.77 (10)**	0.00 (0)
*High and decreasing*	**6.01 (64)**	**91.55 (975)**	**2.44 (26)**
*High and constant*	0.25 (2)	**12.92 (104)**	**86.83 (699)**

Note: Values in bold indicate statistical significance (*p *< .05)

Table 2 also presents the conditional probabilities of Canadians’ compliance levels to staying-at-home during the lifting measures period based on their initial membership during the mandatory measures period. Similarly to social distancing probabilities, Canadians remained stable for the staying-at-home measure during both periods as indicated by the probabilities along the diagonal: 80.24% (*n* = 134) of the population remained in the lowest compliance trajectory; 88.69% (*n* = 910) in the “High and decreasing” trajectory; 85.08% (*n* = 673) in the “High and constant” trajectory. In comparison, few individuals exhibited transition movements between trajectories: 19.76% (*n* = 33) of Canadians previously in the “Low and constant” trajectory are now in the “High and decreasing” trajectory; 8.48% (*n* = 87) of Canadians transitioned from “High and decreasing” to “Low and decreasing”; 2.83% (*n* = 29) shifted from “High and decreasing” to “High and constant”; finally a portion of Canadians (14.92%, *n* = 118) displayed a decrease in compliance levels by shifting from “High and constant” to “ High and decreasing”. Interestingly, we did not find any transition movements between “Low and constant”, “Low and decreasing” and “High and constant”.

**Table 2:** Percentage (and Number) of individuals in each trajectory for staying-at-home measure, by period

	Lifting measures period		
Mandatory measures period	*Low and decreasing*	*High and decreasing*	*High and constant*
*Low and constant*	**80.24 (134)**	**19.76 (33)**	0.00 (0)
*High and decreasing*	**8.48 (87)**	**88.69 (910)**	**2.83 (29)**
*High and constant*	0.00 (0)	**14.92 (118)**	**85.08 (673)**

Note: Values in bold indicate statistical significance (*p *< .05)

### Predicting trajectory membership

We then used the RISK function from PROC TRAJ to investigate the predictive value of social and individual characteristics in predicting trajectory membership. This analysis included controls for age, gender, and level of education, and independent variables were coded as high and low (reference group). Tables 3 and 4 present the odds of factors associated with membership in trajectories for social distancing and staying-at-home measures. 

#### Social distancing

As shown in Table 3, Canadians exhibited greater likelihood of being in the “High and constant” social distancing group compared to the “High and decreasing” social distancing group when possessing high subjective health literacy (*Odds* = 1.67; 95% *CI* [1.17, 2.37]; *p = *.005) during the mandatory measures period. Likewise, they reported greater likelihood when presenting higher perception of provincial social norms (*Odds* = 1.86; 95% *CI* [1.28, 2.70]; *p* <.001), trust in Canadian health services (*Odds* = 1.86; 95% *CI* [1.33, 2.59]; *p* <.001) and clarity (*Odds* = 2.34; 95% *CI* [1.68, 3.26]; *p* <.001). Membership to the “Low and constant” group, in comparison to the “High and decreasing” group, was less likely when reporting higher perception of provincial social norms (*Odds* = 0.27; 95% *CI* [0.09, 0.87]; *p* = .027) and trust in government (*Odds* = 0.19; 95% *CI* [0.07, 0.49]; *p* <.001).

During the lifting period, as outlined in Table 3, Canadians who registered high levels of subjective health literacy (*Odds* = 1.43; 95% *CI* [1.01, 2.04]; *p* = .048), perceived self-efficacy (*Odds* = 1.51; 95% *CI* [1.08, 2.10]; *p* = .017), perceptions of provincial social norms (*Odds* = 1.80; 95% *CI* [1.27, 2.57]; *p* = .001), trust in Canadian health services (*Odds* = 1.54; 95% *CI* [1.08, 2.19]; *p = *.014) and clarity (*Odds* = 2.16; 95% *CI* [1.52, 3.07]; *p* <.001) were more likely associated with the “High and constant” social distancing trajectory when compared to the modal reference group. In comparison to the “High and decreasing group, individuals with high subjective health literacy levels (*Odds* = 0.33; 95% *CI* [0.19, 0.56]; *p* <.001), high trust in Canadian health services (*Odds* =.51; 95% *CI* [0.27, 0.97]; *p* = .038) and high trust in the provincial Prime Minister (*Odds* = 0.45; 95% *CI* [0.26, 0.80]; *p* = .007) are less likely to be found in the “Low and decreasing” trajectory. 

**Table 3:** Factors of trajectory membership for social distancing during the mandatory and lifting measures periods

		Mandatory measures period					Lifting measures period				
Factors	Group (ref group =2)	β	SE	p-value	Odds	Odds 95% CI	β	SE	p-value	Odds	Odds 95% CI
Constant	1	-0.37	0.67	.581	0.69	[0.19, 2.57]	-0.21	0.38	.582	0.81	[0.38, 1.71]
	3	-1.71	0.34	< .001^***^	0.18	[0.09, 0.35]	-2.02	0.35	< .001^***^	0.13	[0.07, 0.26]
Age- Seniors	1	-1.99	1.18	.093.	0.14	[0.01, 1.38]	-1.92	0.55	< .001^***^	0.15	[0.05, 0.43]
	3	0.48	0.16	.003^**^	1.62	[1.18, 2.21]	0.14	0.17	.413	1.15	[0.82, 1.61]
Gender- Male	1	-0.18	0.40	.643	0.84	[0.38, 1.83]	0.72	0.28	.010^*^	2.05	[1.19, 3.56]
	3	-0.73	0.17	< .001^***^	0.48	[0.35, 0.67]	-0.45	0.16	.005^**^	0.64	[0.47, 0.87]
Education Level- University degree	1	-0.07	0.47	.878	0.93	[0.37, 2.34]	-0.56	0.28	.042^*^	0.57	[0.33, 0.99]
	3	-0.04	0.16	.797	0.96	[0.70, 1.31]	0.04	0.16	.807	1.04	[0.76, 1.42]
Subjective Health Literacy-High	1	-0.79	0.43	.063^†^	0.45	[0.20, 1.05]	-1.11	0.27	< .001^***^	0.33	[0.19, 0.56]
	3	0.51	0.18	.005^**^	1.67	[1.17, 2.37]	0.36	0.18	.048^*^	1.43	[1.01, 2.04]
Perceived self-efficacy- High	1	0.17	0.56	.763	1.19	[0.40, 3.55]	0.42	0.31	.170	1.52	[0.83, 2.79]
	3	0.32	0.18	.066^†^	1.38	[0.97, 1.96]	0.41	0.17	.017^*^	1.51	[1.08, 2.10]
Perception of federal norms-High	1	0.32	0.48	.506	1.38	[0.54, 3.53]	-0.02	0.28	.933	0.98	[0.57, 1.70]
	3	0.09	0.20	.662	1.09	[0.74, 1.62]	0.10	0.20	.598	1.11	[0.75, 1.64]
Perception of provincial norms-High	1	-1.30	0.59	.027^*^	0.27	[0.09, 0.87]	-0.33	0.29	.256	0.72	[0.41, 1.27]
	3	0.62	0.18	< .001^***^	1.86	[1.28, 2.70]	0.59	0.18	< .001^***^	1.80	[1.27, 2.57]
Trust in Canadian health services-High	1	-0.15	0.56	.792	0.86	[0.29, 2.58]	-0.68	0.33	.038^*^	0.51	[0.27, 0.97]
	3	0.62	0.17	< .001^***^	1.86	[1.33, 2.59]	0.43	0.18	.014^*^	1.54	[1.08, 2.19]
Trust in the Prime Minister-High	1	-1.66	0.48	< .001^***^	0.19	[0.07, 0.49]	-0.79	0.29	.007^**^	0.45	[0.26, 0.80]
	3	-0.06	0.20	.782	0.94	[0.64, 1.39]	0.03	0.20	.874	1.03	[0.70, 1.53]
Clarity-High	1	-0.82	0.45	.068^†^	0.44	[0.18, 1.06]	-0.17	0.30	.575	0.84	[0.47, 1.52]
	3	0.85	0.17	< .001^***^	2.34	[1.68, 3.26]	0.77	0.18	< .001^***^	2.16	[1.52, 3.07]
Coherence-High	1	0.04	0.41	.914	1.04	[0.47, 2.32]	-0.32	0.31	.298	0.73	[0.40, 1.33]
	3	0.04	0.19	.847	1.04	[0.72, 1.51]	-0.16	0.20	.437	0.85	[0.58, 1.26]

Note: During the mandatory measures period : Trajectory 1= Low and constant; Trajectory 2= High and decreasing; Trajectory 3= High and constant; During the lifting measures period : Trajectory 1= Low and decreasing; Trajectory 2= High and decreasing; Trajectory 3= High and constant

†*p* < .1; *p < .05; **p < .01;**p < .001

#### Staying-at-home

As depicted in Table 4, individuals who indicated higher levels of subjective health literacy (*Odds* = 1.75; *CI* [1.21, 2.54]; *p* = .003), trust in Canadian health services (*Odds* = 1.67; 95% *CI* [1.15, 2.42]; *p* = .006), clarity (*Odds* = 2.23; 95% *CI* [1.53, 3.23]; *p* <.001), and coherence (*Odds* = 1.70; 95% *CI* [1.15, 2.51]; *p* = .008) were more inclined to be associated with the “High and constant” staying-at-home trajectory compared to the “High and decreasing” group during the mandatory measures period. In contrast, Canadians found in the “Low and constant” group were less likely to possess high levels of subjective health literacy (*Odds* = 0.51; 95% *CI* [0.28, 0.93]; *p* = .031), trust in the provincial Prime Minister (*Odds* = 0.39; 95% *CI* [0.21, 0.71]; *p* = .003).

During the lifting period, as presented by Table 4, belonging to the “High and constant” group, as opposed to the “High and decreasing” group, is associated with high levels of subjective health literacy (*Odds* = 1.90; 95% *CI* [1.28, 2.81]; *p* = .001, perception of provincial social norms (*Odds* = 1.63; 95% *CI* [1.10, 2.42]; *p* = .020), trust in Canadian health services (*Odds* = 1.80; 95% *CI* [1.24, 2.62]; *p* = .002) and clarity (*Odds* = 2.05; 95% *CI* [1.39, 3.04]; *p* = .003). On the other hand, Canadians who were less likely to belong in the “Low and decreasing” group recorded high levels of subjective health literacy (*Odds* = 0.46; 95% *CI* [0.26, 0.82]; *p* = .008) and trust in the provincial Prime Minister (*Odds* = 0.38; 95% *CI* [0.20, 0.72]; *p* = .004). 

**Table 4:** Factors of trajectory membership for staying-at-home during the mandatory and lifting measures periods

		Mandatory measures period					Lifting measures period				
Factors	Group (ref group =2)	β	*SE*	*p-value*	*Odds*	*Odds* 95% *CI*	β	*SE*	*p-value*	*Odds*	*Odds* 95% *CI*
Constant	1	-0.80	0.53	.134	0.45	[0.16, 1.27]	-0.82	0.50	.104	0.44	[0.17, 1.17]
	3	-1.58	0.30	< .001^***^	0.21	[0.11, 0.37]	-2.05	0.35	< .001^***^	0.13	[0.06, 0.26]
Age- Seniors	1	-0.68	0.44	.127	0.51	[0.21, 1.20]	-1.27	0.52	.016^*^	0.28	[0.10, 0.78]
	3	0.02	0.17	.885	1.02	[0.73, 1.42]	-0.06	0.18	.737	0.94	[0.66, 1.34]
Gender- Male	1	0.11	0.34	.742	1.12	[0.57, 2.17]	0.87	0.34	.010^*^	2.39	[1.23, 4.65]
	3	-0.70	0.17	< .001^***^	0.50	[0.36, 0.69]	-0.62	0.18	.001^***^	0.54	[0.38, 0.77]
Education Level- University degree	1	0.17	0.36	.633	1.19	[0.59, 2.40]	-0.29	0.33	.377	0.75	[0.39, 1.43]
	3	0.13	0.17	.442	1.14	[0.82, 1.59]	0.01	0.17	.959	1.01	[0.72, 1.41]
Subjective Health Literacy-High	1	-0.68	0.31	.031^*^	0.51	[0.28, 0.93]	-0.77	0.29	.008^**^	0.46	[0.26, 0.82]
	3	0.56	0.19	.003^**^	1.75	[1.21, 2.54]	0.64	0.20	< .001^***^	1.90	[1.28, 2.81]
Perceived self-efficacy- High	1	-0.14	0.35	.701	0.87	[0.44, 1.73]	0.23	0.35	.514	1.26	[0.63, 2.50]
	3	0.06	0.18	.738	1.06	[0.75, 1.51]	0.15	0.18	.396	1.16	[0.82, 1.65]
Perception of federal norms-High	1	0.38	0.40	.331	1.46	[0.67, 3.20]	0.22	0.34	.511	1.26	[0.65, 2.45]
	3	-0.15	0.20	.440	0.86	[0.58, 1.27]	-0.17	0.22	.441	0.84	[0.55, 1.30]
Perception of provincial norms-High	1	-0.56	0.41	.179	0.57	[0.26, 1.28]	-0.21	0.36	.567	0.81	[0.40, 1.64]
	3	0.40	0.21	.062	1.49	[0.99, 2.25]	0.49	0.20	.020^*^	1.63	[1.10, 2.42]
Trust in Canadian health services-High	1	0.17	0.54	.759	1.19	[0.41, 3.42]	-0.75	0.41	.065^†^	0.47	[0.21, 1.06]
	3	0.51	0.19	.006^**^	1.67	[1.15, 2.42]	0.59	0.19	.002^**^	1.80	[1.24, 2.62]
Trust in the Prime Minister-High	1	-0.95	0.31	.003^**^	0.39	[0.21, 0.71]	-0.97	0.33	.004^**^	0.38	[0.20, 0.72]
	3	0.04	0.22	.867	1.04	[0.68, 1.60]	-0.02	0.22	.929	0.98	[0.64, 1.51]
Clarity-High	1	0.44	0.34	.208	1.55	[0.80, 3.02]	-0.10	0.33	.771	0.90	[0.47, 1.73]
	3	0.80	0.19	< .001^***^	2.23	[1.53, 3.23]	0.72	0.20	.003^**^	2.05	[1.39, 3.04]
Coherence-High	1	0.00	0.32	.998	1.00	[0.53, 1.87]	-0.52	0.35	.140	0.59	[0.30, 1.18]
	3	0.53	0.20	.008^**^	1.70	[1.15, 2.51]	0.12	0.22	.578	1.13	[0.73, 1.74]

Note: During the mandatory measures period: Trajectory 1= Low and constant; Trajectory 2= High and decreasing; Trajectory 3= High and constant; During the lifting measures period: Trajectory 1= Low and decreasing; Trajectory 2= High and decreasing; Trajectory 3= High and constant

†*p*< .10; *p < .05; **p < .01; ***p < .001

#### Sociodemographic variables

Age and level of education, except gender, were reported as inconsistent factors of membership to trajectories of social distancing and staying-at-home, as displayed in Tables 3 and 4.

### Predicting trajectory change

We employed multinomial logistic regression to investigate the predictive role of social and individual characteristics in determining trajectory change membership. Three contrasts were deemed feasible for the analysis due to the rare occurrence of trajectory change: “Low and constant” to “Low and decreasing” (0) vs. “Low and decreasing” to “High and decreasing” (1); “High and decreasing” to “Low and decreasing” (0) vs. “High and decreasing” to “High and constant” (1); and “High and constant” to “High and decreasing” (0) vs. remained in “High and constant” (1). Tables 5 and 6 illustrate the results of the multinomial logistic regression in relation to trajectory changes.

#### Social distancing

As shown in Table 5, only a limited number of factors appear to forecast trajectory changes across all contrasts. For instance, Canadians who exhibited high levels of perception of provincial social norms (*Odds* = 4.10; 95% *CI* [1.14, 14.73]; *p* = .030) and trust in Canadian health services (*Odds* = 4.12; 95% *CI* [1.43, 11.88]; *p* = .009) were more likely to transition from “Low and constant” to “High and decreasing” trajectories during the lifting measures period, unlike those who reported high clarity of messages (*Odds* = 0.21; 95% *CI* [0.04, 0.97]; *p = *.046). On the other hand, Canadians were more prone to transition from the “High and decreasing” to the “High and constant” pattern during the lifting period, as opposed to the transition from “High and decreasing” to “Low and decreasing,” if they indicated higher levels of trust in Canadian health services (*Odds* = 2.81; 95% *CI* [1.20, 6.58]; *p = *.018 and higher perception of federal social norms (*Odds* = 2.38; 95% *CI* [0.93, 6.09]; *p* = .070). However, greater perception of provincial social norms (*Odds* = 0.41; 95% *CI* [0.15, 1.10]; *p* = .076) and self-efficacy (*Odds* = 0.48; 95% *CI* [0.21, 1.07]; *p* = .072) reduced the likelihood of transitioning from the “High and decreasing” to the “High and constant” pattern during the transition period.

**Table 5:** Factors of trajectory change for social distancing measure during the first wave

	Comparison 1					Comparison 2					Comparison 3			
Factors	β	*SE*	*p-value*	*Odds*	*Odds* 95% *CI*	β	*SE*	*p-value*	*Odds*	*Odds* 95% *CI*	β	*SE*	*p-value*	*Odds*
Constant	-2.00	0.80	.012^**^	0.14	[0.03, 0.65]	-0.43	0.62	.486	0.65	[0.19, 4.61]	-1.06	0.26	< .001^***^	0.35
Age- Seniors	-0.14	1.01	.892	0.87	[0.12, 6.36]	1.37	0.64	.032^*^	3.92	[1.13, 27.81]	0.14	0.14	.307	1.15
Gender- Male	-0.40	0.54	.460	0.67	[0.23, 1.93]	-0.42	0.41	.300	0.65	[0.29, 4.65]	0.21	0.13	.116	1.23
Education Level- University degree	0.90	0.59	.129	2.45	[0.77, 7.79]	0.83	0.40	.038^*^	2.30	[1.05, 16.30]	0.04	0.13	.769	1.04
Subjective health Literacy- High	-0.55	0.58	.339	0.58	[0.19, 1.78]	-0.06	0.39	.878	0.94	[0.44, 6.68]	0.04	0.16	.811	1.04
Perceived self-efficacy	-0.17	0.50	.741	0.85	[0.31, 2.28]	-0.74	0.41	.072^†^	0.48	[0.21, 3.39]	-0.16	0.14	.276	0.86
Perception of federal norms-High	-0.73	0.64	.254	0.48	[0.14, 1.69]	0.87	0.48	.070^†^	2.38	[0.93, 16.92]	0.01	0.17	.938	1.01
Perception of provincial norms-High	1.41	0.65	.030^**^	4.10	[1.14, 14.73]	-0.90	0.51	.076^†^	0.41	[0.15, 2.90]	-0.10	0.16	.548	0.91
Trust in Canadian health services-High	1.41	0.54	.009^**^	4.12	[1.43, 11.88]	1.03	0.43	.018^*^	2.81	[1.20, 19.93]	0.01	0.15	.931	1.01
Trust in the Prime Minister -High	0.73	0.53	.167	2.08	[0.74, 5.87]	-0.48	0.40	.235	0.62	[0.28, 4.39]	-0.01	0.16	.966	0.99
Clarity-High	-1.58	0.79	.046^*^	0.21	[0.04, 0.97]	0.12	0.38	.757	1.13	[0.53, 7.99]	-0.23	0.15	.141	0.80
Coherence-High	-0.37	0.58	.518	0.69	[0.22, 2.13]	-0.29	0.45	.528	0.75	[0.31, 5.33]	-0.01	0.16	.966	0.99

Note: Comparison 1 = “Low and constant” to “Low and decreasing” (0) vs “Low and constant” to “High and decreasing” (1); Comparison 2 = “High and decreasing” to “Low and constant” (0) vs “High and decreasing” to “High and constant” (1); Comparison 3 = “High and constant” to “High and decreasing” (0) vs remained in “High and constant” during both periods (1)

†*p *< .10; *p < .05; **p < .01; ***p < .001

#### Staying-at-home

Table 6 reveals that shifting from a “Low and constant” to a “High and decreasing” trajectory is more likely when individuals reported high trust in the provincial Prime Minister (*Odds* = 2.08; 95% *CI* [1.08, 4.02]; *p* = .028). Furthermore, an increase in compliance (“High and decreasing” to “High and constant”) as opposed to a decline in compliance (“High and decreasing” to “Low and decreasing”) was more likely when reporting high perception of provincial social norms (*Odds* = 2.22; 95% *CI* [1.12, 4.40]]; *p* = .022). Finally, maintaining membership in “High and constant” trajectories during both periods was more probable when possessing high perception of self-efficacy (*Odds* = 1.30; 95% *CI* [0.98, 1.73]; *p* = .070), as opposed to those reporting high perception of provincial social norms (*Odds* = 0.74; 95% *CI* [0.55, 1.00]; *p* = .051) and trust in Canadian health services (*Odds* = 0.77; 95% *CI* [0.58, 1.02]; *p* = .071).

**Table 6:** Factors of trajectory change for staying-at-home measure during the first wave

	Comparison 1					Comparison 2					Comparison 3				
Factors	β	SE	p-value	Odds	Odds95% CI	β	SE	p-value	Odds	Odds95% CI	β	SE	p-value	Odds	Odds95% CI
Constant	-1.00	0.41	.014^*^	0.37	[0.16, 0.82]	-1.14	0.49	.021^*^	0.32	[0.12, 0.84]	-1.27	0.27	< .001^***^	0.28	[0.16, 0.48]
Age- Seniors	0.07	0.36	.843	1.07	[0.53, 2.18]	-0.37	0.47	.430	0.69	[0.28, 1.73]	0.19	0.15	.198	1.21	[0.91, 1.61]
Gender- Male	-0.46	0.29	.110	0.63	[0.36, 1.11]	-0.10	0.31	.736	0.90	[0.49, 1.66]	0.12	0.13	.346	1.13	[0.87, 1.47]
Education Level- University degree	0.28	0.28	.312	1.32	[0.77, 2.27]	0.22	0.32	.480	1.25	[0.67, 2.33]	-0.07	0.13	.588	0.93	[0.72, 1.20]
Subjective health Literacy- High	0.13	0.27	.621	1.14	[0.67, 1.94]	0.54	0.38	.160	1.71	[0.81, 3.63]	0.00	0.15	.984	1.00	[0.74, 1.35]
Perceived self-efficacy	-0.13	0.29	.647	0.88	[0.50, 1.54]	-0.17	0.32	.601	0.84	[0.45, 1.59]	0.26	0.15	.070^†^	1.30	[0.98, 1.73]
Perception of federal norms-High	-0.40	0.31	.204	0.67	[0.37, 1.24]	-0.26	0.37	.482	0.77	[0.38, 1.59]	-0.14	0.16	.378	0.87	[0.64, 1.18]
Perception of provincial norms-High	0.42	0.31	.172	1.53	[0.83, 2.80]	0.80	0.35	.022^*^	2.22	[1.12, 4.40]	-0.30	0.15	.051^†^	0.74	[0.55, 1.00]
Trust in Canadian health services-High	0.21	0.29	.471	1.23	[0.70, 2.16]	-0.10	0.36	.774	0.90	[0.44, 1.84]	-0.26	0.14	.071^†^	0.77	[0.58, 1.02]
Trust in the Prime Minister -High	0.73	0.33	.028^*^	2.08	[1.08, 4.02]	-0.17	0.38	.648	0.84	[0.40, 1.78]	0.14	0.16	.383	1.15	[0.84, 1.59]
Clarity-High	0.08	0.29	.779	1.08	[0.62, 1.91]	0.59	0.37	.110	1.81	[0.87, 3.76]	0.16	0.16	.297	1.18	[0.87, 1.61]
Coherence-High	-0.49	0.32	.121	0.61	[0.33, 1.14]	-0.23	0.36	.526	0.79	[0.39, 1.62]	0.16	0.17	.356	1.17	[0.84, 1.64]

Note: Comparison 1 = “Low and constant” to “Low and decreasing” (0) vs “Low and constant” to “High and decreasing” (1); Comparison 2 = “High and decreasing” to “Low and decreasing” (0) vs “High and decreasing” to “High and constant” (1); Comparison 3 = “High and constant” to “High and decreasing” (0) vs remained in “High and constant” during both periods (1)

†*p *< .1; *p < .05; **p < .01; ***p < .001

#### Sociodemographic variables

Transitioning from a “High and decreasing” trajectory to a “High and constant” social distancing trajectory was significantly associated with being over 65 years of age (*Odds* = 3.92; *95% CI* [1.13, 13.62]; *p* = .032) and having a university-level education (*Odds* = 2.30; *95% CI* [1.05, 5.04]; *p* = .038).

## Discussion

This study aimed to identify and understand compliance trajectories with preventive measures during the mandatory and lifting measures periods of the COVID-19 pandemic, along with factors associated with these trajectories and with the change of trajectories. In both periods, three distinct compliance trajectories were discerned for social distancing and staying-at-home measures. Notably, a large proportion of Canadians exhibited high compliance, with 93.5% for social distancing and 91.2% for staying-at-home during mandatory measures, and 90.7% for social distancing and 88.3% for staying-at-home during lifting measures. Within the lowest-compliance class, approximately 91% of participants for social distancing and 80% for staying at home remained in the same trajectory class across periods. Health literacy, perception of provincial social norms, trust in Canadian health services, trust in the provincial Prime Minister, and perception of clarity consistently emerged as significant factors for trajectory membership in both preventive measures for both periods. Perceived self-efficacy exclusively predicted membership in social distancing trajectories, while inconsistent predictions were observed for perception of coherence and federal social norms. In terms of trajectory changes, perception of provincial social norms and trust in Canadian health services frequently emerged as significant factors of increasing or maintaining high compliance behaviour. Overall, the study provides valuable insights into the dynamics of compliance with preventive measures, emphasising the role of various factors in shaping individual trajectories and changes in behaviour.

In line with our first objective, we posited that distinct compliance patterns would emerge for both social distancing and staying-at-home measures during the mandatory and lifting periods, with the expectation that a significant majority of Canadians would report high levels of compliance. These initial hypotheses were validated, as our findings unveiled three distinct trajectories during each period. During the mandatory measures period, the lowest compliance trajectory for both social distancing and staying-at-home measures, labelled as *Low and constant*, exhibited stability with an initially lower compliance level. As the measures were lifted, this lowest compliance trajectory underwent a transformation, adopting a declining linear trend. This shift in trend implies that situational factors, such as the easing of measures, might negatively impact the motivation to comply for individuals already at risk of non-compliance. In contrast to findings from other longitudinal studies [2, 14], where the lowest compliance trajectory exhibited a decline from the onset of the pandemic, our trajectory modelling reveals a distinct pattern, a decline in compliance commencing as the measures were lifted for this group. This differentiation is crucial, as it enhances the precision of our understanding of longitudinal compliance patterns by scrutinising specific periods more susceptible to influencing trajectory trends. During both mandatory and lifting measures periods, we observed a group, called *High and decreasing*, that reported a decrease in compliance with an initial high level of compliance with preventive measures. Interestingly, this predominant group, encompassing most Canadians, appeared unaffected by the imposition or lifting of measures; they simply adopted the necessary behaviours and gradually exhibited a waning interest in maintaining these practices. A similar characterisation was noted in previous LCGA studies [1–3, 14]. Existing literature has proposed the concept of pandemic fatigue as a potential explanation for this decline in compliance levels, positing that sustaining beneficial yet demanding behaviours, such as social distancing and staying-at-home, becomes progressively challenging compared to initially adopting them [65]. Given the unconventional nature of the novel preventive measures, it seems justifiable that a single wave of a pandemic may not suffice for complete adoption and perpetuation, as integrating a new behaviour into routine requires time. This aligns with observations in other health-related behaviours, like dietary regimens, fitness programs, or medication plans, where the motivation to uphold costly and unnatural behaviours tends to diminish over time [66]. Lastly, the *High and constant* group exhibited constant patterns of high compliance during both the mandatory and lifting measures periods, seemingly impervious to the changing situation. Overall, our findings align with previous Canadian longitudinal studies on compliance with preventive measures. However, we suggest that the initial decline observed in these studies may not solely be attributed to a changing situation, as hypothesized, but could also be influenced by psychological fatigue, depending on the group.

While executing Latent Class Growth Analysis, we observed variations in the proportion of Canadians in each trajectory between the mandatory and lifting measures periods. For example, regarding the social distancing measure, we identified that 40.57% of individuals were in the *High and constant* trajectory during the mandatory period, but this number decreased to 36.54% during the lifting period. Likewise, in relation to the staying-at-home measure, we noted a decrease in membership for the highest compliance trajectory (*High and constant*), declining from 39.87% during the mandatory period to 35.38% during the lifting period. Given these variations in membership proportions, our second objective sought to describe the transition movements between both periods of the first pandemic wave in terms of group compliance membership. We found that most Canadians preserved a similar pattern of compliance during both periods, as suggested by the probabilities along the diagonals (between 86.83% − 91.55% for social distancing and between 80.24% − 88.69% for staying-at-home). In addition to confirming our initial hypotheses, these findings highlight the importance of the initial compliance level in predicting future compliance patterns, especially in the dynamic context of an evolving pandemic. Indeed, to our knowledge, this is the first study to examine stability within a group of compliance, emphasising the need for governments to implement their interventions in the initial weeks of a health crisis to improve compliance with measures in the long term. Furthermore, this noticeable stability in compliance membership between periods informs us that a change in behaviour is not as affected by situational factors like the easing of preventive measures, but is rather influenced by already present inter-individual differences.

Furthermore, we noticed a general trend where more Canadians transitioned to a lower compliance trajectory rather than high, as the measures were lifted. More specifically, we noted that 12.92% (*n* = 104) of Canadians transitioned from a *High and constant* to a *High and decreasing* social distancing trajectory, and a similar transition was observed for staying-at-home, accounting for 14.92% (*n* = 118). This movement, characterised by a decrease in compliance, represented the most substantial transition between trajectories in our study in terms of number, followed by the transition from *High and decreasing* to *Low and constant.* Therefore, our results suggest that transition movements are more likely to be found within the higher compliance groups, such as *High and constant* and *High and decreasing*. In prior LCGA studies examining compliance with preventive measures [1, 2, 14], the researchers documented fluctuating compliance behaviour during the COVID-19 pandemic across nearly all trajectories, except for the trajectory associated with the highest levels of compliance. This group, identified as *Class 1* in Wright et al. [1], *High and constant* in Courdi et al. [2], and *High adherers* in MacNeil et al. [14], exhibited a consistent compliance level, remaining unaffected by the various waves of the virus. In contrast, the lowest compliance trajectory, designated as *Class 4* in Wright et al. [1], *Fast Decliners* in MacNeil et al. [14], and *Low and fluctuating* in Courdi et al. [2], was characterised by significant variability dependent on the prevailing pandemic situation. However, according to our findings, we identified two distinct forms of variability: one pertains to fluctuations in the trend of a pattern, as exemplified by the change in trend of the lowest compliance trajectory between periods, while the other involves variations in membership within compliance groups, such as the *High and constant* group in our study. This differentiation has not been addressed in studies on compliance behaviour with preventive measures, challenging our conventional perception of stability associated with higher compliance groups. Hence, situational factors, such as the lifting of measures, appear to impact groups disparately, emphasising the importance of elaborating intervention plans tailored to the specific characteristics of each group and not just the lowest trajectory group. Indeed, it allows for targeting individuals at the extremes of compliance behaviour, due to the large sample size of the current study, offering insights not only into groups but also into individuals. Every individual transitioning from a high compliance group to a lower one poses as much of a risk as individuals in the lowest compliance group, as they too are susceptible to finding themselves in these low trajectories, subsequently affecting infection rates.

As mentioned, the proportions along the diagonals in terms of trajectory membership suppose a stability in compliance behaviour throughout the first wave of the pandemic, suggesting that inter-individual differences might be at cause and not situational factors. Our findings consistently demonstrate that health literacy alone serves as a reliable factor of trajectory membership for both social distancing and staying-at-home behaviours across both observed periods. Aligned with existing COVID-19 literature, health literacy—encompassing the ability to comprehend and critically assess health-related information—stands validated as a pivotal individual factor of compliance with preventive measures, particularly amid the challenges posed by the Infodemic [4, 31, 32]. However, contrary to our expectations, health literacy emerged as a factor solely for trajectory membership and not for change in membership. This surprising outcome suggests a certain resilience and stability in the concept of health literacy, impervious to dynamic situations such as the easing of preventive measures. When delving into the factors contributing to a high level of health literacy, it is largely anticipated by constant sociodemographic attributes such as education, income and migration status, as highlighted in the work of Martin et al. [67]. Given the relatively stable nature of these factors and their gradual evolution over an extended period, it could elucidate why health literacy proves to be a more effective factor of the stability of trajectory membership [68]. Our results further imply that health literacy plays a role in shaping the overall compliance pattern within a group characterised by shared stable attributes, as seen in the lowest trajectory groups in our study (*Low and constant* to *Low and decreasing*). Indeed, the probability of belonging in the lowest trajectory significantly decreased when displaying high health literacy levels between the observed periods. With the government initiating the easing of measures, individuals who were less inclined to exhibit high health literacy tended to persist within the same trajectory, but the pattern of their trajectory underwent a shift, adopting a decreasing trend. Therefore, individual factors, such as health literacy, appear to be more adept at forecasting stability within a group while also capturing variations in trends.

We observed another type of variability in our study, that challenged the conventional perception of stability associated with high compliance groups. Trajectory change, observed mostly within the *High and constant* and *High and decreasing* groups, was predicted by social factors, like perception of provincial social norms and trust in Canadian health services. Social norms, defined by a set of rules and expectations in terms of socially acceptable behaviours and attitudes, were often studied in relation to compliance with preventive measures, as they elicit long term changes in behaviour and target large groups [33]. In affirming prior research emphasising the pivotal role of social norms in predicting compliance behaviour [7, 63], our study goes further and proposes that social norms may also serve as a factor for changes in trajectory. In accordance with Reynolds et al.’s comprehensive framework on behaviour change [69], individuals from the same province, acting as referent points, play a crucial role in shaping attitudes and behaviour toward compliance. The salience of this provincial identity may have diminished as the situation evolved with the easing of measures. Notably, during the initial months of the pandemic, most provincial governments conveyed messages of encouragement, emphasising the imperative of collective action within the province to limit the virus’s spread [70, 71]. As the measures were lifted, the salience of this provincial identity waned in the public discourse, potentially explaining the behavioural shifts observed among some Canadians. In contrast, individuals whose provincial identity was initially salient were more likely to comply with the highest compliance trajectory or transition to it even amid the easing of measures. Noteworthy, our results suggest the relevance of the provincial identity in predicting compliance behaviour, compared to national identity, positing that the government should prioritise norm-based interventions at state levels. Furthermore, our study revealed that trust in Canadian health services, rather than trust in the government, emerged as a significant factor of trajectory change, thus affirming our initial hypothesis. Trust in Canadian health services encompasses confidence in the policies implemented and decisions made by the scientific community to safeguard against emerging threats [48]. Throughout the pandemic, scientists were entrusted by governments to devise optimal strategies for curbing the virus’s spread. However, as new information surfaced regarding the virus’s contagiousness, policies underwent adjustments, such as the introduction of mask mandates [17]. Our findings suggest that individuals with higher levels of trust in Canadian health services were not only more likely to comply with recommended behaviour but also demonstrated a propensity to either maintain or transition to higher compliance patterns. This adaptability could stem from their capacity to assimilate novel information about the virus communicated by the scientific community and understand the reasoning behind these policies.

### Strengths and limitations

To our knowledge, this study stands as the first to explore the dynamic nature of behaviour compliance with preventive measures during the COVID-19 pandemic and its associated factors, through Joint-Trajectory analysis. Apart from its innovative methodology, the study boasts several strengths, including its longitudinal design, a representative sample drawn from the Canadian population, short intervals between survey periods during the initial wave, and measurement times spanning both mandatory and lifting measure periods. These attributes collectively enhance the study’s robustness and reliability. However, it is crucial to interpret our results in consideration of several limitations. First and foremost, compliance behaviours such as social distancing and staying-at-home were evaluated through self-reported scores, introducing a potential impact on the reproducibility of our findings. While it is noteworthy that many studies on the COVID-19 pandemic have relied on self-reported compliance measures [1, 2, 14], we advocate for the incorporation of external measures, such as mobile-phone mobility data [65], to mitigate desirability bias. Additionally, our factors were predominantly assessed using single homemade self-reported items, limiting the comprehensive evaluation of the various dimensions inherent in a complex concept like social norms or trust in Canadian health services and challenging the validity of our measures. Furthermore, our operationalisation of health literacy was guided by previous research [2, 4]. In Courdi et al. [2], we focused on a single dimension of the construct, level of understanding, by computing specific items (e.g., “In general, I have a clear understanding of the various measures established by Canada’s public health agency”). In the present study, we extended this approach by capturing all three core components of health literacy rather than one. Nevertheless, even with this broader operationalisation, we acknowledge that our measure does not encompass the full complexity of the health literacy construct. Moreover, we recognise that compliance behaviours, like other behaviours, are influenced by a range of individual factors (e.g., analytical thinking, risk perception, political and worldview orientations, affective responses) and contextual factors (e.g., infection and death rates, severity of imposed restrictions), which were not directly captured in our study. Future research should aim to incorporate a broader set of objective external measures and consider these additional factors to gain a more comprehensive understanding of the underlying mechanisms driving these behaviours. While our measures capture trust in Canadian health services and trust in the provincial Prime Minister’s handling of the crisis, these items may also reflect broader constructs such as trust in public health services and trust in government. Indeed, public health services can be perceived as proxies for scientific expertise, and confidence in the Prime Minister may be taken as an indicator of institutional or political trust. However, we acknowledge that the use of these two single items provides only a partial view and cannot fully represent the complexity of trust in the public health or in governmental institutions. Our operationalisation therefore offers a pragmatic but limited proxy, which future research should expand upon by incorporating multidimensional measures of both scientific and political trust. Lastly, it is essential to exercise caution when interpreting our findings related to factors of trajectory change, as some comparisons involved relatively small sample sizes (e.g., 106 vs. 10).

## Conclusion

Latent Class Growth Analysis proves an innovative and invaluable approach in assessing trends in compliance behaviour with preventive measures, allowing for the identification of inter-individual differences and variability within a group, which was not assessed in previous studies. Through this analysis, we discerned three distinct trajectories during both mandatory and lifting measures periods: Low and constant (decreasing during the lifting measures period), High and decreasing, and High and constant. Expanding our comprehension of compliance behaviour, we delved into transitions between these trajectories and found that the majority of individuals remained within similar compliance groups (e.g., High and constant remained in High and constant during both the mandatory and lifting measures), even amidst the varying context of the pandemic. Contrary to the conventional belief in the stability of high compliance groups, our findings challenge this assumption, revealing that these groups were the most susceptible to transitioning to lower compliance categories. This insight bears significant implications for policies related to compliance behaviour, suggesting that vulnerability to decreased compliance is universal, albeit manifesting differently across various groups. The study also emphasized the importance of prioritizing collaboration during a social crisis to produce relevant research that addresses real-world problems. For example, compliance behaviour played a crucial role in limiting the spread of COVID-19, and understanding the virus’s transmission modes was essential for determining the appropriate preventive measures. This interdependence between these two areas highlights the importance of studying the consequences of a social crisis from an interdisciplinary perspective [72]. In the near future, it would be valuable to explore how to integrate these findings into mathematical modeling of infection diseases, namely compartment models like the Susceptible-Infectious-Recovered (SIR) model, to better inform public health institutions about transmission dynamics.

## Supplementary Information

Supplementary Material 1

## Data Availability

The datasets used and/or analysed during the current study are available from the corresponding author on reasonable request.

## Abbreviations

LCGA: Latent Class Growth Analysis
CNORM: Censored Normal Distribution
BIC: Bayesian Information Criterion
SIR: Susceptible–Infectious–Recovered
