# Supplementary material for: Beyond what meets the eye: unveiling dynamics of compliance with preventive measures in the COVID-19 era
Source: BMC Public Health. 2026 Mar 4;26:1185. doi: 10.1186/s12889-026-26347-y (PMC13067714; doi:10.1186/s12889-026-26347-y)
Supplement: Supplementary file 1 — Supplementary Material 1 [file 12889_2026_26347_MOESM1_ESM.docx]

## Beyond What Meets the Eye: Unveiling Dynamics of Compliance with Preventive Measures in the COVID-19 Era

Sahar Ramazan Ali^1^, Éric Lacourse^2^, Mathieu Pelletier-Dumas^1^, Jean-Marc Lina^3^, Jacques Bélair^4^, Roxane de la Sablonnière^1^

^1^Department of Psychology, Université de Montréal, Montréal, Canada

^2^Department of Sociology, Université de Montréal, Montréal, Canada

^3^Department of Electrical Engineering, École de technologie supérieure, Montréal, Canada

^4^Department of Mathematics, Université de Montréal, Montréal, Canada

*****Corresponding author: Roxane de la Sablonnière

Email: ([roxane.de.la.sablonniere@umontreal.ca](mailto:roxane.de.la.sablonniere@umontreal.ca))

Phone number : 1-514-343-6732

## Supplementary Materials

The present research constitutes a vital component of a comprehensive longitudinal investigation exploring the psychological ramifications of COVID-19 on the Canadian population. Entitled "COVID-19 Canada: the end of the world as we know it?", this project was executed through collaborative efforts involving researchers from diverse disciplines. The overarching aim of this research is to gain profound insights into the multifaceted impacts of the COVID-19 pandemic on the Canadian population, focusing on three pivotal themes: compliance with preventive measures, social cohesion, and overall well-being. Our methodology involved an extensive inquiry into participants' perspectives, encompassing over a hundred variables spanning a spectrum of emotions, behaviors, attitudes, and cognitions related to the ongoing pandemic.

#### **Table 1.** Descriptive Statistics of the Survey

| Time point | | *Survey dates* | *Sample Size (N)* | *Intervals with previous Time Point* | *Number of Weeks Elapsed Since Launch of Survey* | *Attrition Rate* | |
| --- | --- | --- | --- | --- | --- | --- | --- |
| *1* | April 6^th^ – May 6^th^ 2020 | | 3617 |  | 0 | | 0% |
| *2* | April 21^st^ – May 13^th^ 2020 | | 2282 | 2 weeks | 2 | | 36.91% |
| *3* | May 4^th^ – May 25^th^ 2020 | | 2369 | 2 weeks | 4 | | 34.50% |
| *4* | May 18^th^ – June 10^th^ 2020 | | 2296 | 2 weeks | 6 | | 36.52% |
| *5* | June 1^st^ – June 23^rd^ 2020 | | 2154 | 2 weeks | 8 | | 40.45% |
| *6* | June 15^th^ – July 13^th^ 2020 | | 2116 | 2 weeks | 10 | | 41.50% |
| *7* | July 13^th^ – August 8^th^ 2020 | | 2072 | 4 weeks | 14 | | 42.71% |
| *8* | August 17^th^ – September 13^th^2020 | | 1871 | 5 weeks | 19 | | 48.27% |
| *9* | September 21^st^ – October 19^th^ 2020 | | 1821 | 5 weeks | 24 | | 49.65% |
| *10* | November 26^th^ –December 29^th^ 2020 | | 1883 | 9 weeks | 33 | | 47.94% |
| *11* | April 13^th^ – May 31^st^ 2021 | | 2002 | 20 weeks | 53 | | 44.65% |
| *12* | Mars 2^nd^ – April 19^th^ 2022 | | 1672 | 46 weeks | 99 | | 53.77% |

Table 1 illustrates various elements of the survey: the number of time points, the timeline of the survey, and the sample size.

#### **Table 2.** Descriptive Statistics of the Time Points Used for the Study

| Time Point | *Survey Dates* | | *Sample Size (N)* | *Intervals Between Time Points* | *Number of Weeks Elapsed Since Launch of Survey* |
| --- | --- | --- | --- | --- | --- |
| *Mandatory Measure Period* | | | | | |
| *1* | | April 6^th^ – May 6^th^ 2020 | 1984 | 2 weeks | 0 |
| *2* | | April 21^st^ – May 13^th^ 2020 | 1984 | 2 weeks | 2 |
| *3* | | May 4^th^ – May 25^th^ 2020 | 1984 | 2 weeks | 4 |
| *Lifting Measures Period* | | | | | |
| *4* | | May 18^th^ – June 10^th^ 2020 | 1984 | 2 weeks | 6 |
| *5* | | June 1^st^ – June 23^rd^ 2020 | 1984 | 2 weeks | 8 |
| *6* | | June 15^th^ – July 13^th^ 2020 | 1984 | 2 weeks | 10 |

Table 2 presents the coding scheme of the Time Points of this article.

#### **Table 3.** Detailed Description of the Items Used as Dependent Variables

| *Questions from the Survey* | *Scale* | *Dates of Entry in the Survey* |
| --- | --- | --- |
| *Currently, how often do you do the following?* |  |  |
| Maintain a distance of at least two meters (about two arm's lengths) from others when I am not at home | 1-10 (Never to Always) | April 6^th^ – July 13^th^ 2020 |
| Stay home as much as I can | 1-10 (Never to Always) | April 6^th^ – July 13^th^ 2020 |

Participants were questioned on their level of compliance with preventive measures by reporting how often they would follow the public health recommendations.

#### **Table 4.** *Initial* Computation of Social and Individual Factors

| *Factors* | *Questions from the Survey* | *Scale* | *Time Point* |
| --- | --- | --- | --- |
| Subjective Health Literacy | *We would like to assess what Canadians know about COVID-19. Mark whether the following statements are true or false.* |  |  |
|  | COVID-19 is a hoax. | True/False | 1 |
|  | I know what “maintaining a social distance” means. | True/False | 1 |
|  | Avoiding gatherings with large numbers of people helps prevent the spread of COVID-19. | True/False | 1 |
|  | Only the elderly can be seriously affected by COVID-19. | True/False | 1 |
|  | Healthy people should wear facemasks in public to avoid infection. | True/False | 1 |
|  | Staying home if you feel sick is important to prevent the spread of COVID-19. | True/False | 1 |
|  | COVID-19 is no more dangerous than the seasonal flu. | True/False | 1 |
| Perception of Provincial Social Norms | *Using the scale from 1 to 10 below, indicate your level of agreement with each of the statements.* |  |  |
|  | Very few [Your province citizens] support the governmental measures. | 1-10 (Totally disagree and totally agree) | 1 |
|  | In general, [Your province citizens] are complying with the governmental measures. | 1-10 (Totally disagree and totally agree) | 1 |
|  | Most [Your province citizens] agree with the governmental measures. | 1-10 (Totally disagree and totally agree) | 1 |
| Perception of Federal Social Norms | *Using the scale from 1 to 10 below, indicate your level of agreement with each of the statements.* |  |  |
|  | Most Canadians are following governments’ measures recommendations concerning COVID-19. | 1-10 (Totally disagree and totally agree) | 1 |
|  | Most Canadians believe the government measures to fight COVID-19 are appropriate. | 1-10 (Totally disagree and totally agree) | 1 |
| Perceived Self-Efficacy | *Using the scale from 1 to 10 below, indicate your level of agreement with each of the statements.* |  |  |
|  | I do not know what is the best strategy to fight COVID-19. | 1-10 (Totally disagree and totally agree) | 1 |
| Trust | How much do you trust each of the following actors to address the COVID-19 crisis? |  |  |
|  | Canadian hospitals and health services | 1-10 (Totally distrust and totally trust) | 1 |
|  | Prime Minister Justin Trudeau | 1-10 (Totally distrust and totally trust) | 1 |
|  | Premier Minister [Name] [Pipe in the name of the Prime Minister] | 1-10 (Totally distrust and totally trust) | 1 |
|  | Scientists | 1-10 (Totally distrust and totally trust) | 1 |

Computation of Variables

We computed initially the factors (e.g. perception of provincial and federal social norms, trust in Canadian health services and in Prime Minister, perceived self-efficacy, Subjective health literacy, age, education) and dependent variables (compliance with social distancing and stay-at-home) based on our pre-registered framework (see<https://doi.org/10.17605/OSF.IO/R276S>).

After conducting multinomial logistic regression analyses, we identified multicollinearity issues among key factors, namely trust in Prime Minister, trust in Canadian health services, and perception of federal and provincial social norms. This multicollinearity was evident in large standard errors and model instability. For instance, the trust in Canadian health services variable yielded a p-value of 0.798 with standardized errors ranging from [-0.373, 0.289]. Furthermore, the model became unstable when we added or removed factors, resulting in significant coefficient variations. In light of these findings, we revisited the operationalization of our problematic factors to address potential multicollinearity, while keeping the 6 remaining factors and dependent variables unchanged.

Then, we conducted three specific tests to identify the source of the multicollinearity issue: correlation matrices, variance inflation factor (VIF), and condition index (CI). The correlation matrices assess the degree of relationship between pairs of items—higher correlations indicate stronger relationships. The VIF score measures the extent to which the variance of a coefficient is inflated due to multicollinearity. The CI reveals the severity of multicollinearity and identifies which specific combinations of items warrant further investigation. Additionally, we combined the items related to the perception of provincial social norms with those of federal social norms, and items of trust in Canadian health services with those of trust in provincial Prime Minister. This allowed us to examine whether these items could be studied as distinct constructs (e.g., trust in Canadian health services vs. trust in Prime Minister).

Both correlation matrices for social norms and trust variables revealed high correlations, including a 0.63 correlation between items measuring perception of provincial social norms and a 0.61 correlation between items measuring perception of federal social norms, both exceeding the acceptable threshold of 0.50 in Table 6 (Chennamaneni, Echambadi, & Syam, 2016). Additionally, the correlations in Table 6 suggest a potential multicollinearity issue (*r* < 0.50) between the items "Canadian Hospital and Health Services" and "Scientists," as well as between the items "Prime Minister" and "Scientists."

#### **Table 5.** Correlations Between Items of Perception of Provincial and Federal Social Norms

| Items | Very few [Your province citizens] support the governmental measures. (reversed) | In general, [Your province citizens] are complying with the governmental measures. | Most [Your province citizens] agree with the governmental measures. | Most Canadians are following governments’ measures recommendations concerning COVID-19 |
| --- | --- | --- | --- | --- |
| In general, [Your province citizens] are complying with the governmental measures. | 0.36 |  |  |  |
| Most [Your province citizens] agree with the governmental measures. | 0.46 | 0.62 |  |  |
| Most Canadians are following governments’ measures recommendations concerning COVID-19 | 0.22 | 0.44 | 0.39 |  |
| Most Canadians believe the government measures to fight COVID-19 are appropriate | 0.27 | 0.36 | 0.44 | 0.61 |

#### **Table 6.** Correlations Between Items of Trust in Prime Minister and Trust in Canadian Health Services

| Items | Canadian Hospital and health services | Prime Minister | Provincial Minister |
| --- | --- | --- | --- |
| Canadian Hospital and health services |  |  |  |
| Prime Minister | 0.43 |  |  |
| Provincial Minister | 0.31 | 0.27 |  |
| Scientists | 0.62 | 0.45 | 0.32 |

To further assess multicollinearity, we examined the VIFs, finding that none exceeded the threshold of 4 in Tables 7 and 8 (Pan & Jackson, 2008).

#### **Table 7.** Social Norms’ Variance Inflation Factors

| Items | VIF |
| --- | --- |
| Very few [Your province citizens] support the governmental measures | 1.29 |
| In general, [Your province citizens] are complying with the governmental measures | 1.97 |
| Most [Your province citizens] agree with the governmental measures | 1.79 |
| Most Canadians believe the government measures to fight COVID-19 are appropriate | 1.71 |
| Most Canadians are following governments’ measures recommendations concerning COVID-19 | 1.73 |

#### **Table 8.** Trust in Prime Minister and in Canadian Sealth Services’ Variance Inflation Factors

| Items | VIF |
| --- | --- |
| Canadian Hospital and health services | 1.73 |
| Prime Minister | 1.40 |
| Provincial Minister | 1.26 |
| Scientists | 1.74 |

Finally, we examined the condition index (CI) in Tables 9 and 10 to further assess multicollinearity, absent in the VIFs analyses but present in the correlation matrices. According to Liao and Valliant (2012), a CI between 10 and 30 may indicate multicollinearity and warrants further investigation through variance decomposition proportions. If the variance exceeds 0.30, it confirms multicollinearity. Tables 9 and 10 show that all CIs surpassed the threshold of 10. For instance, the item "In general, [Your province citizens] are complying with the governmental measures" had a CI of 13.08, indicating a strong correlation with the item "Very few [Your province citizens] support the governmental measures." (Table 9).

#### **Table 9.** Social Norms’ Condition Index

| Items | Eigenvalue | Condition Index | | Very few [Your province citizens] support the governmental measures | In general, [Your province citizens] are complying with the governmental measures | Most [Your province citizens] agree with the governmental measures | Most Canadians are following governments’ measures recommendations concerning COVID-19 | Most Canadians believe the government measures to fight COVID-19 are appropriate |
| --- | --- | --- | --- | --- | --- | --- | --- | --- |
| Very few [Your province citizens] support the governmental measures | 0.05 | | 10.44 | 0.54 | 0.00 | 0.01 | 0.14 | 0.08 |
| In general, [Your province citizens] are complying with the governmental measures | 0.03 | | 13.08 | 0.31 | 0.30 | 0.16 | 0.05 | 0.11 |
| Most [Your province citizens] agree with the governmental measures | 0.02 | | 15.49 | 0.05 | 0.00 | 0.09 | 0.07 | 0.04 |
| Most Canadians believe the government measures to fight COVID-19 are appropriate | 0.02 | | 16.30 | 0.07 | 0.12 | 0.21 | 0.48 | 0.42 |
| Most Canadians are following governments’ measures recommendations concerning COVID-19 | 0.02 | | 19.41 | 0.02 | 0.57 | 0.52 | 0.24 | 0.34 |

#### **Table 10.** Trust in Prime Minister and in Canadian Health services’ Condition Index

| Items | Eigenvalue | Condition Index | Canadian Hospital and health services | Prime Minister | Provincial Minister | Scientists |
| --- | --- | --- | --- | --- | --- | --- |
| Canadian Hospital and health services | 0.08 | 7.64 | 0.01 | 0.88 | 0.09 | 0.00 |
| Prime Minister | 0.05 | 9.40 | 0.02 | 0.02 | 0.87 | 0.04 |
| Provincial Minister | 0.02 | 15.72 | 0.51 | 0.05 | 0.01 | 0.04 |
| Scientists | 0.01 | 18.50 | 0.45 | 0.05 | 0.03 | 0.92 |

Given that both the correlation matrices and condition indices indicated multicollinearity between the items (trust in Canadian health services and government, perception of provincial and federal social norms), we opted to remove the problematic items for the sake of parsimony.

The final operationalization included single items:

Trust in Canadian health services: "How much do you trust Canadian hospitals and health services to address the COVID-19 crisis?"

Trust in provincial Prime Minister: "How much do you trust the Prime Minister [Name] to address the COVID-19 crisis?"

Perception of federal social norms: "Most Canadians are following governments’ measures recommendations concerning COVID-19."

Perception of provincial social norms: "In general, [Your province citizens] are complying with the governmental measures."

Further attempts to use composite measures, such as exploratory factor analysis and averaging, were problematic. The factor analysis yielded unfavorable results due to high correlations among items, while the averaging approach resulted in large standard errors and p-values close to 1, suggesting multicollinearity again.

#### **Table 11.** *Final* Items Retained for Social and Individual Factors

| *Factors* | *Questions from the Survey* | *Scale* | *Time Point* |
| --- | --- | --- | --- |
| Subjective Health Literacy | *We would like to assess what Canadians know about COVID-19. Mark whether the following statements are true or false.* |  |  |
|  | COVID-19 is a hoax. | True/False | 1 |
|  | I know what “maintaining a social distance” means. | True/False | 1 |
|  | Avoiding gatherings with large numbers of people helps prevent the spread of COVID-19. | True/False | 1 |
|  | Only the elderly can be seriously affected by COVID-19. | True/False | 1 |
|  | Healthy people should wear facemasks in public to avoid infection. | True/False | 1 |
|  | Staying home if you feel sick is important to prevent the spread of COVID-19. | True/False | 1 |
|  | COVID-19 is no more dangerous than the seasonal flu. | True/False | 1 |
| Perception of Provincial Social Norms | *Using the scale from 1 to 10 below, indicate your level of agreement with each of the statements.* |  |  |
|  | In general, [Your province citizens] are complying with the governmental measures. | 1-10 (Totally disagree and totally agree) | 1 |
| Perception of Federal Social Norms | *Using the scale from 1 to 10 below, indicate your level of agreement with each of the statements.* |  |  |
|  | Most Canadians are following governments’ measures recommendations concerning COVID-19. | 1-10 (Totally disagree and totally agree) | 1 |
| Perceived Self-Efficacy | *Using the scale from 1 to 10 below, indicate your level of agreement with each of the statements.* |  |  |
|  | I do not know what is the best strategy to fight COVID-19. | 1-10 (Totally disagree and totally agree) | 1 |
| Trust in Prime Minister | How much do you trust each of the following actors to address the COVID-19 crisis? |  |  |
|  | Premier Minister [Name] [Pipe in the name of the Prime Minister] | 1-10 (Totally distrust and totally trust) | 1 |
| Trust in Canadian Health Services | How much do you trust each of the following actors to address the COVID-19 crisis? | 1-10 (Totally distrust and totally trust) | 1 |
|  | Canadian Hospital and health services | 1-10 (Totally distrust and totally trust) | 1 |
| Clarity | Using the scale from 1 to 10 below, indicate your level of agreement with each of the statements. |  |  |
|  | In general, I have a clear understanding of the various measures established by my provincial public health agency. | 1-10 (Totally disagree and totally agree) | 1 |
| Coherence | Using the scale from 1 to 10 below, indicate your level of agreement with each of the statements. |  |  |
|  | I am confused about the different recommendations coming from the federal and provincial governments and public health agencies. | 1-10 (Totally disagree and totally agree) | 1 |
|  | The measures established by Canadian and provincial public health agencies are similar. | 1-10 (Totally disagree and totally agree) | 1 |

#### **Table 12.** Final Computation of Factors (High vs Low)

| *Factors* | First Quartile (Lower or Qqual) | % (n) |
| --- | --- | --- |
| *Subjective Health Literacy* | 0.8 |  |
| *High* |  | 70.36 (1396) |
| *Low* |  | 29.64 (588) |
| *Perception of Provincial Social Norms* | 7 |  |
| *High* |  | 51.61 (1024) |
| *Low* |  | 32.61 (647) |
| *Perception of Federal Social Norms* | 6 |  |
| *High* |  | 71.22 (1413) |
| *Low* |  | 28.78 (571) |
| *Perceived Self-Efficacy* | 5 |  |
| *High* |  | 60.64 (1203) |
| *Low* |  | 31.25 (620) |
| *Trust in provincial Prime Minister* | 6 |  |
| *High* |  | 71.32 (1415) |
| *Low* |  | 28.68 (569) |
| *Trust in Canadian Health Services* | 8 |  |
| *High* |  | 56.00 (1111) |
| *Low* |  | 44.00 (873) |
| *Clarity* | 8 |  |
| *High* |  | 59.53 (1181) |
| *Low* |  | 40.47 (803) |
| *Coherence* | 6.5 |  |
| *High* |  | 30.04 (1388) |
| *Low* |  | 69.96 (596) |

#### **Table 13.** Descriptive Statistics of Covariables

| *Factors* | % (*n*) |
| --- | --- |
| *Gender* |  |
| *Male* | 50.96 (1011) |
| *Female* | 49.04 (973) |
| *Age* |  |
| *65 and over* | 73.89 (1466) |
| *Between 18 and 64* | 26.11 (518) |
| *Level of Education* |  |
| *University Level* | 52.87 (1049) |
| *Non-University Level* | 47.13 (935) |

#### **Table 14.** Bayesian Information Criterion for Selection of a Trajectory Model for Social Distancing During the Mandatory Measures Period

| *Model* | *K* | *Order* | *BIC* |
| --- | --- | --- | --- |
| *1* | 1 | 1 | -8119.41* |
| *2* | 2 | 1,1 | -7625.68* |
| *3* | 3 | 1,1,1 | -7505.66* |
| ***4*** | **3** | **0,1,1** | **-7501.67*** |
| *5* | 3 | 0,1,0 | -7510.15 |

Table 14 shows the various attempts at obtaining the best model for social distancing measure during the mandatory measures period. Only models with an * were retained for comparison, as the parameters’ estimates were significant (*p* < .05). The models were compared according to the Bayesian information criterion (BIC): indeed, the solution with the lowest absolute value of BIC was selected. In this case, we selected the model with 3 trajectories (0,1,1).

#### **Table 15.** Bayesian Information Criterion for Selection of a Trajectory Model for Social Distancing During the Lifting Measures Period

| *Model* | *K* | *Order* | *BIC* |
| --- | --- | --- | --- |
| *1* | 1 | 1 | -9323.48* |
| *2* | 2 | 1,1 | -8738.45* |
| *3* | 3 | 1,1,1 | -8412.58 |
| ***4*** | **3** | **1,1,0** | **-8414.28*** |
| *5* | 3 | 0,1,0 | -8426.06 |

Table 15 shows the various attempts at obtaining the best model for social distancing measure during the lifting measures period. Only models with an * were retained for comparison, as the parameters’ estimates were significant (*p* < .05). The models were compared according to the Bayesian information criterion (BIC): indeed, the solution with the lowest absolute value of BIC was selected. In this case, we selected the model with 3 trajectories (1,1,0).

#### **Table 16.** Bayesian Information Criterion for Selection of a Trajectory Model for Staying at Home During the Mandatory Measures Period

| *Model* | *K* | *Order* | *BIC* |
| --- | --- | --- | --- |
| *1* | 1 | 1 | -8114.43* |
| *2* | 2 | 1,1 | -7742.22* |
| *3* | 3 | 1,1,1 | -7584.12 |
| *4* | 3 | 1,1,0 | -7597.97* |
| ***5*** | **3** | **0,1,0** | **-7594.57*** |

Table 16 shows the various attempts at obtaining the best model for staying at home measure during the mandatory measures period. Only models with an * were retained for comparison, as the parameters’ estimates were significant (*p* < .05). The models were compared according to the Bayesian information criterion (BIC): indeed, the solution with the lowest absolute value of BIC was selected. In this case, we selected the model with 3 trajectories (0,1,0).

#### **Table 17.** Bayesian Information Criterion for Selection of a Trajectory Model for Staying at Home During the Lifting Measures Period

| *Model* | *K* | *Order* | *BIC* |
| --- | --- | --- | --- |
| *1* | 1 | 1 | -9810.18* |
| *2* | 2 | 1,1 | -9405.57* |
| *3* | 3 | 1,1,1 | -9184.62* |
| ***4*** | **3** | **1,1,0** | **-9182.35*** |
| *5* | 3 | 0,1,0 | -9189.48* |

Table 17 shows the various attempts at obtaining the best model for staying at home measure during the lifting measures period. Only models with an * were retained for comparison, as the parameters’ estimates were significant (*p* < .05). The models were compared according to the Bayesian information criterion (BIC): indeed, the solution with the lowest absolute value of BIC was selected. In this case, we selected the model with 3 trajectories (1,1,0).
